# Rhinoceros Serum microRNAs: Identification, Characterization, and Evaluation of Potential Iron Overload Biomarkers

**DOI:** 10.3389/fvets.2021.711576

**Published:** 2021-12-16

**Authors:** Jessye Wojtusik, Erin Curry, Terri L. Roth

**Affiliations:** Center for Conservation and Research of Endangered Wildlife (CREW), Cincinnati Zoo and Botanical Garden, Cincinnati, OH, United States

**Keywords:** biomarker, endangered species, iron overload disorder, microRNA, rhinoceros, serum

## Abstract

Iron overload disorder (IOD) in critically endangered Sumatran (*Dicerorhinus sumatrensis*) and black (*Diceros bicornis*) rhinoceros is an over-accumulation of iron in organs which may exacerbate other diseases and indicate metabolic disturbances. IOD in rhinos is not well understood and diagnostics and therapeutics are limited in effectiveness. MicroRNAs (miRNAs) are small non-coding RNAs capable of altering protein synthesis. miRNA expression responds to physiological states and could serve as the basis for development of diagnostics and therapeutics. This study aimed to identify miRNAs differentially expressed among healthy rhinos and those afflicted with IOD or other diseases (“unhealthy”), and assess expression of select miRNAs to evaluate their potential as biomarkers of IOD. miRNAs in serum of black (*n* = 11 samples; five individuals) and Sumatran (*n* = 7 samples; four individuals) rhinos, representing individuals categorized as healthy (*n* = 9), unhealthy (*n* = 5), and afflicted by IOD (*n* = 3) were sequenced. In total, 715 miRNAs were identified, of which 160 were novel, 131 were specific to black rhinos, and 108 were specific to Sumatran rhinos. Additionally, 95 miRNAs were specific to healthy individuals, 31 specific to unhealthy, and 63 were specific to IOD individuals. Among healthy, unhealthy, and IOD states, 21 miRNAs were differentially expressed (*P* ≤ 0.01). Five known miRNAs (let-7g, miR-16b, miR-30e, miR-143, and miR-146a) were selected for further assessment *via* RT-qPCR in serum from black (*n* = 61 samples; seven individuals) and Sumatran (*n* = 38 samples; five individuals) rhinos. let-7g, miR-30e, and miR-143 all showed significant increased expression (*P* ≤ 0.05) during IOD (between 1 and 2 years prior to death) and late IOD (within 1 year of death) compared to healthy and unhealthy individuals. miR-16b expression increased (*P* ≤ 0.05) in late IOD, but was not different among IOD, healthy, and unhealthy states (*P* > 0.05). Expression of miR-146a increased in IOD and late IOD as compared to unhealthy samples (*P* ≤ 0.05) but was not different from the healthy state (*P* > 0.05). Selected serum miRNAs of black and Sumatran rhinos, in particular let-7g, miR-30e, and miR-143, could therefore provide a tool for advancing rhino IOD diagnostics that should be further investigated.

## Introduction

The survival of all five extant species of rhinoceros (henceforth referred to as “rhinos”) is threatened due to several challenges, including poaching and habitat loss. Although some populations are experiencing an increase in numbers, the global rhino population has recently been in decline. Four of the species (black (*Diceros bicornis*), greater one-horned (GOH; *Rhinoceros unicornis*), Sumatran (*Dicerorhinus sumatrensis*), and white (*Ceratotherium simum*) rhinos) are managed in *ex situ* populations which may provide insurance against species extinction. However, maintaining genetically sound and sustainable populations is not without challenges. Experience in animal husbandry and advances in veterinary care have led to improved longevity and productivity for intensively managed rhinos. However, certain diseases, seemingly unique to individuals in human care, impact overall population well-being.

Iron overload disorder (IOD) is an abnormal over-accumulation of iron as hemosiderosis in organs, particularly in the liver and spleen of rhinos (1), associated with increased inflammation and possibly a consequence of metabolic disturbances (2, 3). Iron is an essential nutrient; however, as no physiological mechanism exists to rid the body of any excess, other than hemorrhage, maintaining iron homeostasis is a complex process. In rhinos, the mechanisms regulating iron homeostasis, and consequently the causes of IOD, are not fully understood. Genetics, metabolism, and nutrition may all play a role (2, 4, 5). Despite widespread efforts to manage IOD in zoo-housed rhinos, IOD is frequently reported as a comorbidity.

Sumatran and black rhinos appear to be the most susceptible to developing IOD; however, there are limited reports of IOD in GOH (1, 6). White rhinos do not appear to be afflicted. IOD in living rhinos is difficult to diagnose. In early stages animals may exhibit few clinical signs, if any, and in later stages, clinical signs are not specific to the disease; therefore, IOD is generally confirmed post-mortem (7). Additionally, IOD may exacerbate and potentially be masked by other diseases (8). The search for reliable IOD biomarkers for rhinos has traversed broad studies of genetics, metabolomics, microbiomics, and concentrated on specific proteins, oxidative markers and beyond (2–4, 9–12), shedding light on many aspects of this disorder, but also creating new questions and revealing areas in need of further research. Therefore, it is important to continue to investigate and develop new and innovative methods to aid in its diagnosis and treatment.

MicroRNAs (miRNAs) are small (~18–22 nucleotides) non-coding RNAs that regulate gene expression post-transcriptionally to influence various biological processes including cell proliferation, apoptosis, metabolism, and response to disease (13). Found throughout the body, in both intra- and extra-cellular environments, miRNAs exert an effect by influencing protein synthesis and can be used as biomarkers for physiological status and various disease states (14, 15). In humans and mice, miRNAs have been successfully used to diagnose viral infections including Ebola (16) and hepatitis (17), and various cancers (18), among a variety of other diseases. Studies focused on the use of miRNAs in both diagnostics and therapeutics are increasing rapidly (19), including those centered specifically on diseases of the liver (20, 21). As miRNAs have been shown to regulate expression of genes involved in iron acquisition, export, storage, and utilization (22) as well as iron overload in humans (23), potential exists for the development of miRNA-based diagnostics and therapeutics for IOD in rhinos.

The study of miRNAs in wildlife species has not progressed as quickly as that in human or lab animal species. However, Murchison et al. (24) found significant differences in miRNA expression in facial tumors compared to healthy tissue of Tasmanian devils (*Sarcophilus harrisii*), and it may be possible to diagnose elephant endotheliotropic herpesvirus through detection of viral miRNAs in infected animals (25). Due to their abundance and ubiquitous role in regulating physiological processes, miRNAs have the potentital to be useful in wildlife species management. miRNAs could be used to diagnose and monitor various physiological states including growth and development, reproductive status, and multiple diseases, including IOD, or in the development of targeted treatments. The main goals of this study were to (1) identify miRNAs differentially expressed between healthy rhinos and those known to be afflicted with IOD or other diseases, and (2) assess longitudinal expression of select miRNAs to evaulate their potential as IOD biomarkers.

## Materials and Methods

All samples were previously banked and used opportunistically in this study with permission from each owning institution. Individual rhinos (*n* = 13) were maintained at North American institutions (*n* = 6) at the time of sample collection. Samples were stored at −20 and/or −80°C for 1–24 years (AVG: 12.4 ± 1.2) and each had experienced one to two freeze-thaw cycles prior to use in this project.

### miRNA Sequencing

A total of 18 serum samples was submitted for sequencing. Nine samples from healthy black (*n* = 5) and Sumatran (*n* = 4) rhinos were compared to four samples collected from IOD animals (*n* = 3 individuals; one black rhino, two Sumatran rhinos) and five samples collected from unhealthy individuals known to be diagnosed with another disease including cancer, arthritis, and hypercalcemia (four black rhinos, one Sumatran rhino). Serum samples from IOD (diagnosis confirmed at necropsy) and unhealthy (diagnosed with another disease at the time of collection) individuals were sequenced simultaneously with healthy samples. IOD and unhealthy individuals acted as their own internal controls, represented during a healthy period of their lifetime and again when known to be in a diseased state. The black rhino with IOD contributed three samples: one healthy sample collected 5 years prior to death, one sample collected 14 weeks prior to death, and another collected 9 days prior to death. Unhealthy animals were included to distinguish between miRNAs indicative of a non-specific disease state and those specific to IOD. The availability of samples from both species was limited due to population size. Individuals were considered “healthy” based on two main criteria: (1) there were no known reports of illness within ± 1 year of sample collection and (2) the individual did not die within the following 5 years. Samples were shipped on dry-ice to LCSciences (Houston, TX) for sequencing.

Total RNA was extracted using the Plasma/Serum RNA Purification Midi Kit (Norgen Biotek, Thorold, Ontario) according to manufacturer's instructions. The total RNA quality and quantity were analyzed using Bioanalyzer 2100 (Agilent 2100 Bioanalyzer Instrument, RRID:SCR_019389) with RIN number >7.0. TruSeq Small RNA Sample Prep Kits (Illumina, San Diego, CA) were used as per manufacturer's protocol to generate a small RNA library from approximately 1 μg of total RNA from each sample. Single-end sequencing 50 bp was performed on Illumina HiSeq 2500 (Illumina HiSeq 2500 System, RRID:SCR_016383) following vendor's recommended protocol.

### Sequencing Bioinformatics and Statistical Analysis

Small RNA sequencing raw data were filtered using an in-house program, ACGT101-miR (LC Sciences), to remove low quality reads, 3' adapter sequences, contaminations, and repeats. Sequences of 18–26 nucleotides were annotated in the Rfam database [ver.13.0; (26)] to remove other non-coding RNA (rRNA, tRNA, snRNA, snoRNA) and degraded fragments of mRNA. Remaining unique sequences were aligned against the miRBase microRNA database [ver. 22.0; (27); miRBase, RRID:SCR_003152]. As miRNAs have not been sequenced for any rhinoceros species, sequences were mapped to other mammalian species including, but not limited to, *Bos taurus* (*bta*), *Homo sapien* (*hsa*), and *Mus musculus* (*mmu*) in miRBase 22.0 by BLAST search to identify potential known miRNAs and novel 3p- and 5p- derived miRNAs. Length variation at both 3' and 5' ends and one mismatch inside of the sequence were allowed in the alignment. The sequences mapping to specific mature miRNAs in hairpin arms were identified as known miRNAs.

Normalization of sequence counts was performed by LCSciences. Sequence counts of individual miRNAs were divided by the median value of the ratio between the count of a specific sample and a pseudo-reference sample. The pseudo-reference sample was the count geometric mean of all samples. Differential expression of normalized values was assessed by analysis of variation. Sequencing data were presented as mean normalized count ± SEM and significance was defined as *P* ≤ 0.01. All sequence information was submitted to miRBase for inclusion into the database.

### RT-qPCR

Five miRNAs, found to be differentially expressed among the healthy, unhealthy, and IOD states *via* sequencing, bta-let-7g_R-1 (let-7g; TGAGGTAGTAGTTTGTACAGT), bta-miR-16b_R+1 (miR-16b; TAGCAGCACGTAAATATTGGCG), bta-miR-30e-5p (miR-30e; TGTAAACATCCTTGACTGGAAGCT), bta-miR-143_R-2 (miR-143; TGAGATGAAGCACTGTAGCT), and mmu-miR-146a-5p_R+1 (miR-146a; TGAGAACTGAATTCCATGGGTTA), were selected for further assessment *via* RT-qPCR to confirm expression patterns observed *via* sequencing and to further define expression patterns over time. All selected miRNAs were expressed by all sequenced individual rhinos and selection criteria included a *P* ≤ 0.01 and a fold change difference of >2X when average normalized IOD expression, as determined by sequencing, was compared to both healthy and unhealthy averages.

Additional miRNAs that showed the least amount of variation in abundance among all sequenced samples, bta-let-7c-5p (TGAGGTAGTAGGTTGTATGGTT) and bta-miR-331-5p_R+3 (TCTAGGTATGGTCCCAGGGAT), were selected as housekeeper controls. Serum samples from seven black and five Sumatran rhinos (*n* = 99: 61 black and 38 Sumatran rhino samples; 4–15 samples per individual) were shipped to Qiagen (Germantown, MD) for RT-qPCR analysis. Samples were selected to represent healthy (*n* = 80), unhealthy (*n* = 7), and IOD (*n* = 12) individuals within both species and were equally distributed between sexes when possible. For each individual, samples spanned multiple years so that expression over time could be assessed. Due to limited sample volume, it was not possible to submit the same serum sample for both sequencing and PCR in many cases; instead, a sample with a similar date (generally ± 1 month) was used instead.

RNA was extracted from serum samples and was reverse-transcribed (RT) into cDNA then run in duplicate on Qiagen's miRCURY LNA miRNA PCR Custom panel assays. Melting curves were generated for each reaction to confirm a single peak with expected Tm which indicated a single specific product was amplified. A “no template” sample was included as a negative control and Qiagen's RNA spike-in kit was used to generate technical controls for RNA isolation, to detect differences in extraction efficiency, and for cDNA synthesis, to evaluate the RT reaction.

For the RT-qPCR data, normalization using global mean was performed based on the average of the assays detected in all samples (28). The normalized Cq values were calculated as follows: normalized Cq = global mean Cq (sample 1)–assay Cq (miRNA of interest in sample 1). These values were used in calculating relative expression. Concentrations from the same individual within a state were averaged prior to assessment, so that no individual was overrepresented. Descriptive statistics including mean, standard de*via*tion, and standard error of the mean, were calculated for healthy, unhealthy and IOD states. As it is difficult to ascertain when IOD begins, the IOD state was further divided into IOD (>1 year but <2 years prior to death; *n* = 5) and late IOD (≤1 year prior to death; *n* = 7). Of 99 serum samples assessed, there were previously generated serum ferritin concentration data for 84 samples (*n* = 54 black rhino and 30 Sumatran rhino samples; previously published) (11). miRNA expression among states (healthy, unhealthy, IOD and late IOD) was compared using analysis of variance with *post-hoc* Bonferroni for pairwise comparison. These data were also used in correlation analysis between ferritin concentrations and expression of let-7g and miR-146a, as both miRNAs have an established relationship with ferritin in other species (29, 30). RT-qPCR statistical significance was defined as *P* ≤ 0.05. Statistical analysis was performed using SPSS for Windows (Version 27; IBM Corp. Armonk, NY; BDSC Cat#7008, RRID:BDSC_7008). Data is presented as mean ± SEM.

## Result

In total, 715 miRNAs, ranging in length from 18 to 26 nucleotides, were identified in black (*n* = 607 miRNAs) and Sumatran (*n* = 584 miRNAs) rhino serum (Supplementary Figure 1; range: 311–439 miRNAs per individual). All data are available in an open access repository (31). When compared to the miRBase database, 160 miRNAs did not match any previously reported sequences in any species and therefore are reported as novel. Of the total miRNAs identified, 131 (75 known, 56 novel) were specific to black rhinos and 108 (84 known, 24 novel) were specific to Sumatran rhinos. None of the miRNAs found to be specific to the Sumatran rhino group were expressed in more than three of the seven sequenced samples. One miRNA, bta-mir-2369-p3_1ss12TA, specific to black rhinos, was found in all 11 black rhino serum samples while an additional five miRNAs (bta-miR-208a_R+1, and four novel miRNAs) were found in 9–10 of 11 black rhino samples. The remaining miRNAs specific to black rhinos were expressed in fewer than five of the 11 sequenced black rhino samples.

**Figure 1:**
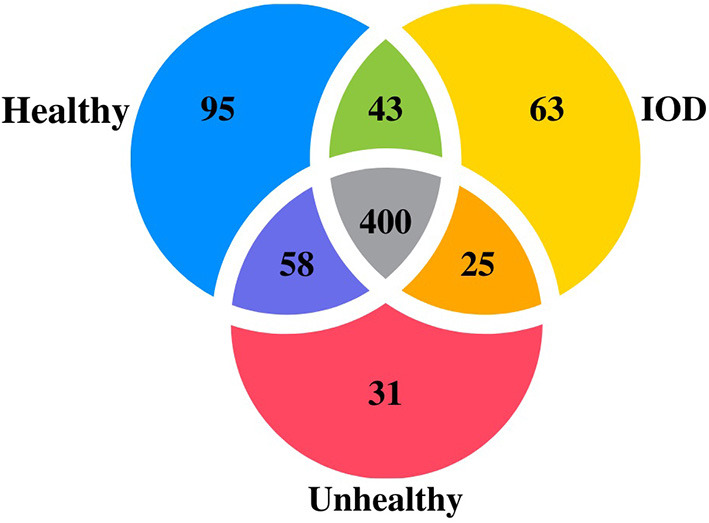
Venn diagram depicting number of microRNAs expressed in serum from healthy (*n* = 9), unhealthy (*n* = 5) and IOD (n = 4 samples; three individuals) afflicted black (*Diceros bicornis*) and Sumatran (*Dicerorhinus sumatrensis*) rhinos.

Additionally, 95 miRNAs (52 known, 43 novel) were specific to healthy individuals, 31 miRNAs (23 known, eight novel) specific to unhealthy, and 63 miRNAs (48 known, 15 novel) were specific to IOD individuals (Figure 1). Twenty-one miRNAs were found to be differentially expressed among the health status states at a *P* ≤ 0.01 (Table 1).

**Table 1:** Differentially expressed serum miRNA normalized sequence count means of healthy (*n* = 9), unhealthy (*n* = 5), and IOD (*n* = 4) afflicted black (*Diceros bicornis*) and Sumatran (*Dicerorhinus sumatrensis*) rhinos (*P* ≤ 0.01).

**miRNA name**	**Sequence**	***P* value**	**Mean expression value**		
			**Healthy**	**Unhealthy**	**IOD**
bta-let-7g_R-1	TGAGGTAGTAGTTTGTACAGT	<0.001	555.7^a^	447.5^a^	1284.4^b^
bta-let-7i_R-1	TGAGGTAGTAGTTTGTGCTGT	<0.001	309.9^a^	412.6^a^	1003.9^b^
bta-miR-1842_L+1R-2_1ss10TC	TTGGCTCTGCGAGGTCGGCT	<0.001	37.8^a^	96.7^b^	46.7^a^
bta-miR-30e-5p	TGTAAACATCCTTGACTGGAAGCT	<0.001	507.1^a^	404.5^a^	1352.0^b^
hsa-miR-148b-5p_L+1	GAAGTTCTGTTATACACTCAGGC	0.003	0.8^a^	0.5^a^	8.5^b^
hsa-miR-486-3p_R-1	CGGGGCAGCTCAGTACAGGA	0.003	31.6^a^	45.1^a^	232.6^b^
bta-miR-7_R-4	TGGAAGACTAGTGATTTTGT	0.003	6.6^a^	13.9^ab^	29.7^b^
mmu-let-7j_1ss8TG	TGAGGTAGTAGTTTGTGCTGTTAT	0.004	1.1^ab^	1.0^a^	5.5^b^
eca-miR-95_R-1	TTCAACGGGTCTTTATTGAGC	0.004	0.7^a^	6.5^ab^	20.6^b^
bta-miR-146a_R-2_1ss18AG	TGAGAACTGAATTCCATGGGTT	0.004	1190.5^a^	1314.0^a^	4598.1^b^
bta-miR-16b_R+1	TAGCAGCACGTAAATATTGGCG	0.006	1430.4^a^	1459.9^a^	7607.8^b^
bta-miR-143_R-2	TGAGATGAAGCACTGTAGCT	0.007	228.9^a^	135.6^a^	1286.1^b^
PC-3p-41523_54^*^	CCCTGAACTAGGAGTCTGGAGT	0.007	0.3^a^	3.0^a^	3.2^a^
bta-miR-214_L+1R-3	TACAGCAGGCACAGACAGGC	0.007	207.4^a^	74.9^a^	71.7^a^
efu-miR-9277_L+3	TCGAATCCTGCCGACTACGC	0.007	2.5^ab^	12.9^b^	0.0^a^
hsa-miR-320b_R-1	AAAAGCTGGGTTGAGAGGGCA	0.008	1.3^a^	6.7^ab^	8.4^b^
bta-miR-378_R+1	ACTGGACTTGGAGTCAGAAGGCT	0.01	8.3^a^	23.3^ab^	34.0^b^
hsa-miR-378a-5p	CTCCTGACTCCAGGTCCTGTGT	0.01	0.0^a^	0.5^a^	1.4^a^
mmu-miR-146a-5p_R+1	TGAGAACTGAATTCCATGGGTTA	0.01	23.5^a^	9.5^a^	143.0^b^
bta-miR-362-5p_R-2	AATCCTTGGAACCTAGGTGTGA	0.01	0.2^a^	0.0^a^	2.0^a^
hsa-miR-106b-3p_R-2	CCGCACTGTGGGTACTTGCT	0.01	12.0^a^	21.1^a^	47.4^a^

*P-value indicates significant difference among the three health states as determined by analysis of variance. Specific differences between each state, as determined by post-hoc Bonferroni test, are indicated by different superscript letters. Novel miRNAs are indicated with a*

* *following the name*.

**Figure 2:**
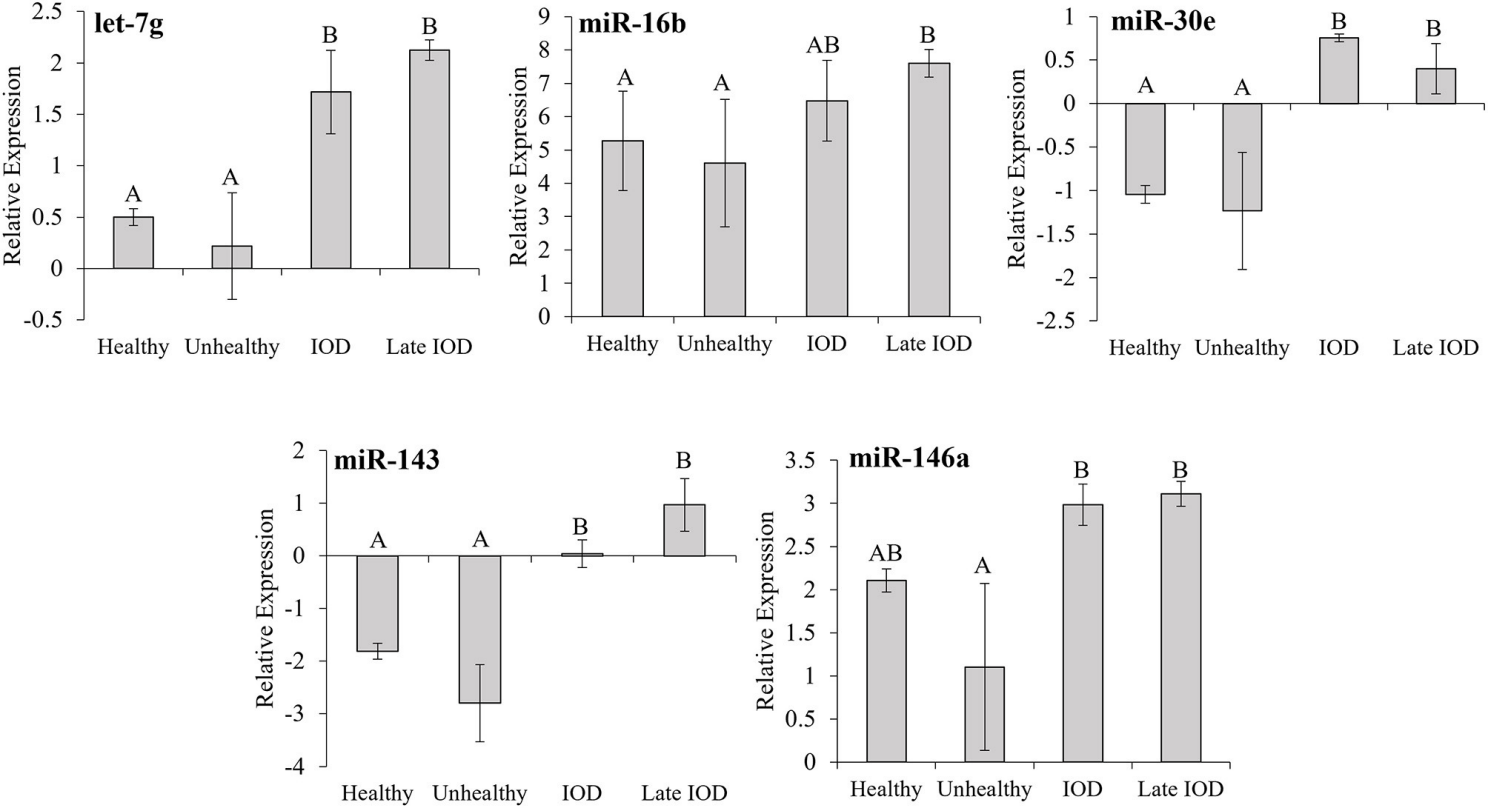
Average normalized relative expression of miRNAs let-7g, miR-16b, miR-30e, miR-143, and miR-146a in healthy (*n* = 80), unhealthy (*n* = 7), and IOD afflicted (*n* = 12) black (*Diceros bicornis*) and Sumatran (*Dicerorhinus sumatrensis*) rhinos as determined by RT-qPCR. Relative expression was determined following normalization using a global mean with consideration of house-keeping miRNAs. IOD afflicted samples were separated into two groups: IOD (>1 year but <2 years prior to death; *n* = 5) and late IOD (≤1 year prior to death; *n* = 7). Data represent mean ± SEM. Different letters indicate statistical differences among states within each miRNA expression group (*P* ≤ 0.05).

Five known miRNAs, found to be differentially expressed among the healthy, unhealthy, and IOD states *via* sequencing, let-7g, miR-16b, miR-30e, miR-143, and miR-146a, were selected for further assessment *via* RT-qPCR (Figure 2). RT-qPCR analysis revealed let-7g, miR-30e, and miR-143 all showed significant increases (*P* ≤ 0.05) in expression during IOD (any sample between 1 and 2 years out from date of death) and late IOD (any sample within 1 year of death) as compared to healthy and unhealthy individuals. miR-16b expression increased (*P* ≤ 0.05) in late IOD, but there was no statistical difference among IOD, healthy and unhealthy states (*P* > 0.05). Expression of miR-146a increased in IOD and late IOD as compared to unhealthy samples (*P* ≤ 0.05) but was not statistically different from the healthy state (*P* > 0.05).

Correlation analysis between miR-146a expression and serum ferritin concentrations revealed a strong negative relationship for black rhinos (r = −0.79; *P* < 0.001) and a marginal positive relationship for Sumatran rhinos (r = 0.46; *P* = 0.01). No meaningful correlation existed between serum ferritin concentrations and let-7g expression for neither black (r = −0.29; *P* = 0.01) nor Sumatran (r = 0.18; *P* = 0.33) rhinos.

## Discussion

Five promising IOD biomarkers in black and Sumatran rhinos have been identified and characterized, providing a new venue with potential to enhance diagnostics for this disease while offering possible targets for therapeutic treatments and study in other taxonomic groups afflicted by IOD.

The total number of miRNAs discovered in black and Sumatran rhino serum is comparable to what has been reported in serum of other species (32, 33). Due to the potential diversity of miRNAs in various tissues that were not included in this study, it is important to note that the discovered miRNAs do not represent all rhino miRNAs. The number of miRNAs specific to black or Sumatran rhinos was surprising as miRNAs are generally highly conserved among mammalian species. However, miRNA expression can be altered by geographical location, diet, exercise, age and metabolic and reproductive states (14, 15, 34–36). Therefore, differences among individudal miRNA expression are not uncommon. Furthermore, most of the miRNAs classified as species-specific were found in only a few individuals. Only one, miR-2369-p3-1ss12TA, was found in all black rhinos sampled and none of the Sumatran rhino samples. Evaluation of a greater sample size is required before this miRNA can be considered species-specific.

Based on the sequencing data, five miRNAs (let-7g, miR-16b, miR-30e, miR143, and miR-146a) were identified as potential biomarkers of IOD in rhinos as their expression was significantly higher in samples from rhinos afflicted with IOD than from healthy and unhealthy samples. Expression of these miRNAs was further assessed in 99 serum samples (61 from black rhinos and 38 from Sumatran rhinos) *via* RT-qPCR revealing a similar pattern to the sequencing data: each of the five targeted miRNAs increased in IOD and late IOD, though the change was most pronounced in miRs 143, 30e, and let-7g. Although miR-16b and 146a expression also increased during IOD affliciton, differential expression among health status states may not be adequate for these miRNAs to act as sole biomarkers. Yet, these miRNAs may be useful in a panel of multiple markers. Due to limited availability of IOD positive samples, this study could not conclusively establish the efficacy of all five miRNAs as potential biomarkers, but it does reveal their promise as candidates.

Homologous sequences of all five miRNA have been identified in over 30 species and each has hundreds of potential targets and functions (27, 37). As this study focused on the relationship of miRNAs with iron overload, IOD-associated physiological mechanisms are discussed in detail but do not encompass all potential targets or roles for these miRNAs.

### let-7g

let-7g is a tumor suppressing miRNA that has an antioxidative and anti-inflammatory effect in endothlial cells of mice (38). let-7g expression was greater during IOD and late IOD as compared to healthy and unhealthy levels (*P* ≤ 0.05). IOD in captive black rhinos is associated with increased inflammation (2, 3), therefore the increase in let-7g expression demonstrated during IOD may occur to limit IOD-associated inflammation or to mitigate oxidative stress resulting from excessive iron. In humans, let-7g may be regulated by the heavy chain subunit of ferritin, an iron storage protein responsible for iron uptake and release (29). Ferritin is often used as an indicator of iron overload in humans; however, there is debate over the usefulness of ferritin as an IOD biomarker in rhino species (10, 11). In this study, correlation analysis between let-7g and ferritin concentrations in black rhinos (11) and Sumatran rhinos (10) did not reveal strong correlations (r = −0.29 and r = 0.18, respectively). It has been previously reported that ferritin concentrations in rhinos are associated with factors other than iron status (10, 11).

### miR-16b

miRNAs within the miR-16 cluster play a regulatory role in iron homeostasis and liver diseases in humans (17, 39). miR-16 expression increases during hepatitis C infection and non-alcoholic fatty liver disease (17) and regulates hepatocyte apoptosis during liver failure (40). miR-16s regulate iron homeostasis *via* inhibition of divalent metal transporter 1 (DMT1) which is responsible for iron uptake and delivery (39). In this study, miR-16b expression was greater in late IOD samples (*P* < 0.05) as compared to healthy and unhealthy samples, but was not statistically different among IOD, healthy, and unhealthy states (*P* > 0.05). miR-16b may be increased in response to iron overload in rhinos in an effort to limit any further absorption of iron from the diet. However, since a significant rise does not appear to occur until late stage IOD, miR-16b is likely not adequate for diagnostic purposes, unless considered alongside other markers.

### miR-30e

miR-30e was expressed at higher levels in IOD and late IOD states than both healthy and unhealthy states (*P* ≤ 0.05). The miR-30 family members (a–e) are involved in a number of physiological processes including cell differentiation and apoptosis, and also have a role in a number of diseases including mammary and liver neoplasia (41, 42). miR-30e expression, specifically, is significantly reduced in hepatocellular carcinoma and may be a useful biomarker for this disease in humans (42). The relationship between miR-30s and iron status is not well defined. miR-30b expression and total iron content were greater in preeclampsia placental tissue than normal placental tissue (43). It was suggested that overexpression of miR-30b resulted in an increase in cellular iron which promoted ferroptosis (43), an iron-dependent form of cell death (44). Ferroptosis has been linked with iron-overload and liver injury in humans and mice (45, 46), though its role in rhino IOD has not been investigated. It is possible that the significant increase in miR-30e expression found in IOD-afflicted rhinos may be associated with ferroptosis.

### miR-143

miR-143 expression was greater in rhinos presenting with IOD in both early and late stages as compared to healthy and unhealthy animals (*P* ≤ 0.05) and has good potential as an IOD biomarker. A known tumor-suppressor, miR-143 promotes apoptosis *via* the p53 pathway (47). As p53 plays a significant role in iron homeostasis (48) and induces ferroptosis, miR-143 may regulate IOD in rhinos *via* this same pathway. In obese mice, miR-143 expression is increased and this up-regulation appears to be associated with glucose metabolism and reduced insulin sensitivity (49). It has been suggested that IOD in captive black rhinos is a consequence of decreased insulin sensitivity or disturbances in glucose metabolism (2). Therefore, the IOD-associated increase in miR-143 expression could be indicative of these metabolic changes.

### miR-146a

miR-146a has been shown to play a prominent role in the regulation of the immune system in both humans and mice (50). In the IOD state, miR-146a expression was not statistically different from the healthy state (*P* > 0.05) and therefore, not likely to be as useful as a sole biomarker for IOD in rhinos. However, numerically, expression was higher in the IOD state and may approach significance with additional data. In women and diabetic patients, miR-146a expression was negatively correlated with ferritin values (30). Ferritin is commonly used as marker of iron status; however, in overweight and obese individuals, ferritin primarily serves as a marker of inflammation (51). In rhinos, fluctuations in ferritin concentrations do not necessarily reflect iron status; ferritin has been shown to be greater in healthy individuals than those with IOD in some cases (11). It is possible that ferritin concentrations are primarily associated with inflammation in rhinos, thereby confounding interpretation of ferritin data in relation to IOD. The black rhino data reported herein revealed a similar relationship to that in women and diabetic humans: a moderate negative correlation (r = −0.79) between serum ferritin concentrations and miR-146a expression in matched samples. Additional research is needed before a full understanding of the interplay of ferritin, miR-146a, insulin status, and IOD can be defined. Correlation analysis between Sumatran rhino ferritin values and miR-146a expression contrastingly demonstrated a slight postive relationship (r = 0.46). This species difference is somewhat unexpected, but evidence from studies investigating fecal metabolome and ferritin in both species suggest that the pathophysiology of IOD may differ between black and Sumatran rhinos (10–12).

### Limitations

Samples were collected opportunistically and not specifically for the purposes of this study. As such, there are a number of limitations that may have impacted the outcomes of this study and should be considered during the interpretation of data. Due to the rarity of the species investigated, sample sizes were limited which likely impacted the statistical power of the dataset. Specifically, expression data found to be insignificant may have approached significance with more data. The stability of miRNAs under certain storage conditions may also be of concern. miRNAs remain stable in storage at temperatures ranging from −20 to −80°C (52) and through at least four to eight freeze thaw cycles (53, 54); however, there are mixed results regarding the impact of storage time, with one study finding no impact after 17 years in storage (54) but another finding a significant impact after 10 years (52). The samples used herein were limited to one to two freeze-thaw cycles prior to use and stored at −20 and/or −80°C; however, some were stored for up to 24 years. Unfortunately, as each animal served as its own control and transitioned from a healthy to a diseased state, there is no way to accurately separate the impact of time from disease within this study. Furthermore, IOD is a progressive disease and as there is currently no definitive early diagnostic tests, it is possible individuals described as healthy may have been accumulating iron at the time of collection. Without an accurate diagnostic test, this limitation is unavoidable; however, to limit the impact, individuals selected for inclusion in the healthy state were not displaying clinical signs of any disease state at time of collection and samples were collected at least 5 years prior to death. The authors recognize these limitations and the possible impact they may have on the interpretation of the data and recommend further investigations into the role of miRNAs in IOD.

## Conclusion

Much remains unknown about the etiology and progression of IOD in rhinos; thus examining physiological changes associated with this disease is crucial for the advancement of diagnostics and treatments. This characterization of serum miRNAs in rhinos provides valuable insight into differentially expressed miRNAs. With further study, these miRNAs may prove useful as biomarkers, individually or as a panel, for diagnostic tests for IOD in rhinos, or as targets for specifically designed treatments.

## Data Availability Statement

The datasets presented in this study can be found in online repositories. The names of the repository/repositories and accession number(s) can be found below: https://data.mendeley.com/datasets/9wxcd3t3tt/4.

## Ethics Statement

Ethical review and approval was not required for the animal study because all rhinoceros serum samples were collected opportunistically as part of veterinary evaluations or as by-products of training activities prior to and unrelated to this study. No samples were collected specifically for this study.

## Author Contributions

JW, EC, and TR contributed to study design, securing funding for project completion, contributed to editing the manuscript, and approved of the submitted version. JW lead project completion with input and guidance from EC and TR completed the statistical analysis, and wrote the first draft of the manuscript. All authors contributed to the article and approved the submitted version.

## Funding

Funding for this study was provided by the Eppley Foundation, and the PI was supported by a generous gift from an anonymous private donor supplemented by a private gift from Elizabeth Tu Hoffman.

## Conflict of Interest

The authors declare that the research was conducted in the absence of any commercial or financial relationships that could be construed as a potential conflict of interest.

## Publisher's Note

All claims expressed in this article are solely those of the authors and do not necessarily represent those of their affiliated organizations, or those of the publisher, the editors and the reviewers. Any product that may be evaluated in this article, or claim that may be made by its manufacturer, is not guaranteed or endorsed by the publisher.
