# Supplementary material for: Rhinoceros Serum microRNAs: Identification, Characterization, and Evaluation of Potential Iron Overload Biomarkers
Source: Front Vet Sci. 2021 Dec 16;8:711576. doi: 10.3389/fvets.2021.711576 (PMC8716540; doi:10.3389/fvets.2021.711576)
Supplement: Supplementary file 1 [file Table_1.DOCX]

Supplementary Material

# Supplementary Data

**Supplementary Figure 1.** Serum microRNA normalized sequence count means ± SEM of black (*Diceros bicornis;* n = 11) and Sumatran (*Dicerorhinus sumatrensis;* n = 7) rhinos.

|  |  | **Species** | |
| --- | --- | --- | --- |
| **miRNA name** | **Sequence** | **Black** | **Sumatran** |
| bta-miR-1 | TGGAATGTAAAGAAGTATGTAT | 10.4 ± 5.7 | 15.9 ± 8.4 |
| bta-let-7f | TGAGGTAGTAGATTGTATAGTT | 195.4 ± 29.6 | 322.7 ± 58.7 |
| mmu-let-7f-1-3p_R-1 | CTATACAATCTATTGCCTTCC | --- | 1.3 ± 0.9 |
| bta-let-7e_R+1 | TGAGGTAGGAGGTTGTATAGTT | 6.3 ± 2.0 | 21.2 ± 6.0 |
| bta-let-7a-3p_R-1 | CTATACAATCTACTGTCTTT | 6.3 ± 1.3 | 6.4 ± 2.9 |
| bta-miR-7_R-4 | TGGAAGACTAGTGATTTTGT | 18.3 ± 5.3 | 8.8 ± 2.1 |
| bta-let-7i_R-1 | TGAGGTAGTAGTTTGTGCTGT | 527.9 ± 129.3 | 531.5 ± 106.9 |
| mml-let-7i-3p_L+1R-1 | CTGCGCAAGCTACTGCCTTGC | 15.5 ± 3.9 | 9.8 ± 4.8 |
| bta-let-7b_R-2 | TGAGGTAGTAGGTTGTGTGG | 840.9 ± 209.2 | 757.7 ± 88.4 |
| hsa-let-7b-3p_R-1 | CTATACAACCTACTGCCTTCC | 14.3 ± 3.7 | 11.2 ± 4.5 |
| bta-let-7g_R-1 | TGAGGTAGTAGTTTGTACAGT | 633.5 ± 127.2 | 824.9 ± 149.3 |
| hsa-let-7g-3p_R-3 | CTGTACAGGCCACTGCCT | --- | 0.2 ± 0.2 |
| bta-let-7c | TGAGGTAGTAGGTTGTATGGTT | 13.7 ± 2.4 | 23.7 ± 3.8 |
| bta-let-7d_R-2 | AGAGGTAGTAGGTTGCATAG | 100.9 ± 18.5 | 137.6 ± 21.7 |
| hsa-let-7d-3p_R-2 | CTATACGACCTGCTGCCTTT | 70.5 ± 13.3 | 88.3 ± 25.3 |
| mdo-let-7f-2-3p_1ss22CT | CTATACAGTCTACTGTCTTTCT | 0.4 ± 0.3 | --- |
| hsa-miR-7-1-3p_1ss22AT | CAACAAATCACAGTCTGCCATT | 2.9 ± 0.8 | 1.0 ± 0.8 |
| bta-let-7a-5p | TGAGGTAGTAGGTTGTATAGTT | 232.6 ± 38.0 | 367.6 ± 69.0 |
| hsa-miR-9-5p | TCTTTGGTTATCTAGCTGTATGA | --- | 1.7 ± 1.7 |
| hsa-miR-9-3p | ATAAAGCTAGATAACCGAAAGT | 0.3 ± 0.3 | 0.8 ± 0.7 |
| bta-miR-10a_R-1 | TACCCTGTAGATCCGAATTTGT | 490.9 ± 93.7 | 575.9 ± 118.5 |
| hsa-miR-10a-3p_R-1 | CAAATTCGTATCTAGGGGAAT | --- | 0.2 ± 0.2 |
| bta-miR-10b_R-1 | TACCCTGTAGAACCGAATTTGT | 1104.8 ± 264.7 | 1851.8 ± 404.7 |
| bta-miR-15a | TAGCAGCACATAATGGTTTGT | 439.8 ± 116.3 | 248.7 ± 42.9 |
| mmu-miR-15a-3p_2ss10CT22AT | CAGGCCATATTGTGCTGCCTCT | 1.4 ± 0.8 | 1.9 ± 1.3 |
| bta-miR-15b_R-3 | TAGCAGCACATCATGGTTT | 630.8 ± 162.2 | 304.5 ± 38.7 |
| hsa-miR-15b-3p_R-3 | CGAATCATTATTTGCTGCT | 59.5 ± 15.6 | 32.5 ± 8.8 |
| chi-miR-16a-3p_L-9R+1 | CCAGTATTAACTGTGCTGCT | 0.5 ± 0.3 |  |
| bta-miR-16b_R+1 | TAGCAGCACGTAAATATTGGCG | 3441.7 ± 1686.6 | 1560.6 ± 422.2 |
| hsa-miR-16-2-3p_L+1R-2_1ss11CT | ACCAATATTATTGTGCTGCTT | 5.2 ± 2.7 | 9.6 ± 4.7 |
| bta-miR-17-5p_R-3 | CAAAGTGCTTACAGTGCAGGT | 298.4 ± 111.6 | 126.8 ± 29.1 |
| bta-miR-17-3p | ACTGCAGTGAAGGCACTTGT | --- | 0.2 ± 0.2 |
| bta-miR-18b_R-4 | TAAGGTGCATCTAGTGCA | 1.7 ± 1.1 | 0.1 ± 0.1 |
| bta-miR-18a_R-2 | TAAGGTGCATCTAGTGCAGA | 47.6 ± 19.0 | 24.5 ± 6.1 |
| mmu-miR-18a-3p_R-1 | ACTGCCCTAAGTGCTCCTTCT | 4.4 ± 1.7 | 3.0 ± 2.2 |
| bta-miR-19b_R-2 | TGTGCAAATCCATGCAAAACT | 322.2 ± 57.6 | 183.3 ± 56.6 |
| bta-miR-19a_R-2 | TGTGCAAATCTATGCAAAACT | 146.8 ± 29.3 | 82.8 ± 28.5 |
| hsa-miR-20b-5p | CAAAGTGCTCATAGTGCAGGTAG | 1.7 ± 0.8 | --- |
| bta-miR-20a_R-2 | TAAAGTGCTTATAGTGCAGGT | 403.8 ± 131.4 | 147.7 ± 35.0 |
| bta-miR-21-5p_R-3 | TAGCTTATCAGACTGATGTTG | 3466.1 ± 766.2 | 3130.2 ± 515.4 |
| bta-miR-21-3p_L+1R-2 | CAACAGCAGTCGATGGGCTGT | 40.8 ± 10.2 | 49.4 ± 9.2 |
| bta-miR-22-5p_R-2 | AGTTCTTCAGTGGCAAGCTT | 99.5 ± 34.5 | 82.3 ± 20.8 |
| bta-miR-22-3p_R-2 | AAGCTGCCAGTTGAAGAAC | 4749.1 ± 1122.2 | 4177.1 ± 318.8 |
| hsa-miR-23a-5p_R+2 | GGGGTTCCTGGGGATGGGATTTGC | 71.7 ± 17.2 | 28.8 ± 8.5 |
| bta-miR-23a_R-1 | ATCACATTGCCAGGGATTTCC | 1275.5 ± 152.6 | 821.9 ± 88.8 |
| bta-miR-23b-5p_R-1 | GGGTTCCTGGCATGCTGATT | 0.4 ± 0.4 | --- |
| bta-miR-23b-3p_R-5 | ATCACATTGCCAGGGATT | 163.4 ± 38.3 | 110.5 ± 14.4 |
| bta-miR-24-3p_R-2 | TGGCTCAGTTCAGCAGGAAC | 2144.1 ± 138.6 | 1851.6 ± 353.7 |
| hsa-miR-24-2-5p_L+1R+1 | GTGCCTACTGAGCTGAAACACAGT | 33.2 ± 7.8 | 23.3 ± 7.0 |
| hsa-miR-25-5p | AGGCGGAGACTTGGGCAATTG | 0.3 ± 0.3 | --- |
| bta-miR-25 | CATTGCACTTGTCTCGGTCTGA | 1552.4 ± 474.7 | 678.5 ± 123.0 |
| bta-miR-26a | TTCAAGTAATCCAGGATAGGCT | 525.1 ± 82.3 | 680.2 ± 96.0 |
| bta-miR-26b_R-1 | TTCAAGTAATTCAGGATAGGT | 534.2 ± 80.1 | 602.9 ± 68.1 |
| hsa-miR-26b-3p | CCTGTTCTCCATTACTTGGCT | 5.6 ± 2.1 | 5.4 ± 3.1 |
| bta-miR-27b | TTCACAGTGGCTAAGTTCTGC | 1400.2 ± 130.7 | 1084.8 ± 87.9 |
| bta-miR-27a-5p_R-1 | AGGGCTTAGCTGCTTGTGAGC | 152.7 ± 35.6 | 228.2 ± 113.0 |
| bta-miR-27a-3p | TTCACAGTGGCTAAGTTCCG | 2308.6 ± 376.2 | 2876.5 ± 706.0 |
| bta-miR-28 | AAGGAGCTCACAGTCTATTGAG | 9.7 ± 2.2 | 10.1 ± 2.2 |
| hsa-miR-28-3p_R+1 | CACTAGATTGTGAGCTCCTGGAA | 73.2 ± 17.7 | 75.9 ± 14.1 |
| mmu-miR-29a-5p_R-2 | ACTGATTTCTTTTGGTGTTC | 0.3 ± 0.3 | --- |
| bta-miR-29a_L-1R-3 | TAGCACCATCTGAAATCGG | 3098.3 ± 662.5 | 3619.1 ± 901.1 |
| mdo-miR-29b-2-5p_L-1R-4 | CTGGTTTCACATGGTGGCT | --- | 2.5 ± 1.7 |
| bta-miR-29b_R-4 | TAGCACCATTTGAAATCAG | 122.5 ± 28.0 | 106.8 ± 36.8 |
| bta-miR-29d-5p_R-1 | TGACCGATTTCTCCTGGTGT | 1.9 ± 0.9 | 0.8 ± 0.6 |
| bta-miR-29c_R-3 | TAGCACCATTTGAAATCGG | 207.9 ± 32.4 | 257.7 ± 81.4 |
| bta-miR-30b-5p | TGTAAACATCCTACACTCAGCT | 854.5 ± 193.3 | 672.1 ± 204.2 |
| bta-miR-30e-5p | TGTAAACATCCTTGACTGGAAGCT | 659.0 ± 176.5 | 729.6 ± 195.9 |
| hsa-miR-30e-3p_1ss22CT | CTTTCAGTCGGATGTTTACAGT | 13.7 ± 3.0 | 24.4 ± 3.9 |
| bta-miR-30c_R+1 | TGTAAACATCCTACACTCTCAGCT | 309.1 ± 67.5 | 387.8 ± 102.4 |
| bta-miR-30d_R-4 | TGTAAACATCCCCGACTGGA | 639.1 ± 135.3 | 605.3 ± 91.0 |
| bta-miR-30a-5p_R-4 | TGTAAACATCCTCGACTGGA | 218.6 ± 38.6 | 416.1 ± 34.6 |
| hsa-miR-30a-3p | CTTTCAGTCGGATGTTTGCAGC | 5.9 ± 1.8 | 20.9 ± 4.9 |
| bta-miR-31 | AGGCAAGATGCTGGCATAGCT | 10.6 ± 6.9 | 1.5 ± 0.9 |
| hsa-miR-32-5p_R-2 | TATTGCACATTACTAAGTTG | 46.7 ± 11.8 | 25.1 ± 11.2 |
| bta-miR-33a_R-3 | GTGCATTGTAGTTGCATT | 0.6 ± 0.3 | 1.6 ± 0.8 |
| hsa-miR-33a-3p_R-3 | CAATGTTTCCACAGTGCAT | --- | 0.1 ± 0.1 |
| bta-miR-34a_R-3 | TGGCAGTGTCTTAGCTGGT | 80.1 ± 27.8 | 34.6 ± 4.0 |
| bta-miR-34c_R+1 | AGGCAGTGTAGTTAGCTGATTGC | --- | 0.2 ± 0.2 |
| bta-miR-92b_R-2 | TATTGCACTCGTCCCGGCCT | 19.4 ± 6.1 | 32.5 ± 17.9 |
| bta-miR-92a | TATTGCACTTGTCCCGGCCTGT | 3576.8 ± 800.3 | 1970.9 ± 136.0 |
| bta-miR-93_R-1 | CAAAGTGCTGTTCGTGCAGGT | 1576.7 ± 486.8 | 616.2 ± 136.4 |
| hsa-miR-93-3p_R+1 | ACTGCTGAGCTAGCACTTCCCGA | 9.3 ± 3.1 | 7.4 ± 3.5 |
| eca-miR-95_R-1 | TTCAACGGGTCTTTATTGAGC | 10.9 ± 4.9 | 1.0 ± 0.8 |
| bta-miR-96_R-4 | TTTGGCACTAGCACATTTT | 4.3 ± 1.7 | 9.4 ± 4.7 |
| bta-miR-98_R-1 | TGAGGTAGTAAGTTGTATTGT | 17.1 ± 4.6 | 37.0 ± 8.3 |
| hsa-miR-98-3p_R-3 | CTATACAACTTACTACTTT | --- | 0.2 ± 0.2 |
| bta-miR-99a-5p | AACCCGTAGATCCGATCTTGT | 1126.6 ± 286.5 | 1423.7 ± 217.8 |
| bta-miR-99a-3p_R+2 | CAAGCTCGCTTCTATGGGTCT | --- | 0.4 ± 0.3 |
| bta-miR-99b_R-1 | CACCCGTAGAACCGACCTTGC | 124.0 ± 24.1 | 190.2 ± 15.0 |
| hsa-miR-101-5p_L+1R-2 | TCAGTTATCACAGTGCTGATG | 1.2 ± 0.8 | 0.8 ± 0.6 |
| bta-miR-101_R+1 | TACAGTACTGTGATAACTGAAT | 192.0 ± 54.4 | 326.6 ± 41.7 |
| hsa-miR-103a-2-5p_R-2 | AGCTTCTTTACAGTGCTGCCT | --- | 0.7 ± 0.5 |
| bta-miR-103_R-5 | AGCAGCATTGTACAGGGC | 351.2 ± 53.8 | 402.5 ± 18.7 |
| bta-miR-106b | TAAAGTGCTGACAGTGCAGAT | 551.8 ± 132.1 | 488.5 ± 206.3 |
| hsa-miR-106b-3p_R-2 | CCGCACTGTGGGTACTTGCT | 19.7 ± 6.4 | 31.2 ± 11.7 |
| bta-miR-122_R-1 | TGGAGTGTGACAATGGTGTTT | 623.8 ± 366.9 | 2976.0 ± 816.1 |
| hsa-miR-122-3p_R-1 | AACGCCATTATCACACTAAAT | 1.1 ± 1.1 | 1.3 ± 0.9 |
| bta-miR-124a_R-2 | TAAGGCACGCGGTGAATGCCA | --- | 1.1 ± 1.1 |
| bta-miR-125b_R-2 | TCCCTGAGACCCTAACTTGT | 174.2 ± 28.3 | 186.8 ± 22.3 |
| hsa-miR-125b-2-3p_L-2 | ACAAGTCAGGCTCTTGGGAC | 0.3 ± 0.3 | 2.6 ± 1.4 |
| bta-miR-125a_R-1 | TCCCTGAGACCCTTTAACCTGT | 185.5 ± 26.4 | 249.2 ± 32.5 |
| bta-miR-126-5p | CATTATTACTTTTGGTACGCG | 627.8 ± 142.2 | 541.4 ± 83.7 |
| bta-miR-126-3p_L+1 | TCGTACCGTGAGTAATAATGCG | 1138.0 ± 234.3 | 1083.0 ± 152.9 |
| bta-miR-127_R-4 | TCGGATCCGTCTGAGCTT | 1.0 ± 0.7 | 2.5 ± 1.7 |
| bta-miR-128_R-2 | TCACAGTGAACCGGTCTCT | 98.7 ± 11.8 | 81.6 ± 8.3 |
| bta-miR-129-3p | AAGCCCTTACCCCAAAAAGCAT | 0.3 ± 0.3 | --- |
| hsa-miR-130b-5p_R-2 | ACTCTTTCCCTGTTGCACT | 0.2 ± 0.2 | 0.5 ± 0.4 |
| bta-miR-130b_R-1 | CAGTGCAATGATGAAAGGGCA | 50.6 ± 12.4 | 13.4 ± 4.5 |
| bta-miR-130a_R-2 | CAGTGCAATGTTAAAAGGGC | 47.0 ± 5.8 | 96.7 ± 21.0 |
| hsa-miR-132-5p | ACCGTGGCTTTCGATTGTTACT | --- | 0.6 ± 0.6 |
| bta-miR-132 | TAACAGTCTACAGCCATGGTCG | 1.8 ± 1.0 | 0.4 ± 0.3 |
| bta-miR-133b_R-1 | TTTGGTCCCCTTCAACCAGCT | 743.8 ± 723.5 | 49.5 ± 18.9 |
| hsa-miR-133a-5p_R-4 | AGCTGGTAAAATGGAACC | --- | 1.3 ± 1.0 |
| hsa-miR-136-3p_L-1 | ATCATCGTCTCAAATGAGTCT | 0.1 ± 0.1 | 1.5 ± 1.2 |
| bta-miR-138 | AGCTGGTGTTGTGAATCAGGCCG | 0.2 ± 0.2 | 9.9 ± 4.1 |
| hsa-miR-138-1-3p_R-1_1ss21CT | GCTACTTCACAACACCAGGGT | --- | 0.3 ± 0.3 |
| bta-miR-139 | TCTACAGTGCACGTGTCTCCAGT | 34.4 ± 5.9 | 61.2 ± 4.7 |
| hsa-miR-140-5p | CAGTGGTTTTACCCTATGGTAG | 2.1 ± 0.8 | 2.3 ± 0.9 |
| bta-miR-140_L-1R+1 | ACCACAGGGTAGAACCACGGAC | 228.7 ± 58.0 | 337.4 ± 125.3 |
| bta-miR-141_R-1 | TAACACTGTCTGGTAAAGATG | 12.5 ± 4.3 | 27.9 ± 15.0 |
| bta-miR-142-5p_L+2R-2 | CCCATAAAGTAGAAAGCACT | 1406.4 ± 258.8 | 2203.1 ± 467.1 |
| bta-miR-142-3p_L+2R-2 | GTAGTGTTTCCTACTTTATGGA | 69.1 ± 21.0 | 62.0 ± 23.0 |
| hsa-miR-143-5p_R-1 | GGTGCAGTGCTGCATCTCTGG | --- | 0.7 ± 0.6 |
| bta-miR-143_R-2 | TGAGATGAAGCACTGTAGCT | 215.9 ± 53.5 | 827.1 ± 458.3 |
| hsa-miR-144-5p_R+1 | GGATATCATCATATACTGTAAGT | 2.9 ± 1.4 | 15.5 ± 3.7 |
| bta-miR-144_R-3 | TACAGTATAGATGATGTAC | 496.9 ± 91.1 | 1183.4 ± 305.6 |
| bta-miR-145_R-4 | GTCCAGTTTTCCCAGGAAT | 412.0 ± 106.9 | 440.6 ± 74.6 |
| mml-miR-145-3p_L-2 | ATTCCTGGAAATACTGTTCTT | 21.3 ± 7.3 | 62.6 ± 25.5 |
| bta-miR-146a_R-2_1ss18AG | TGAGAACTGAATTCCATGGGTT | 2118.7 ± 877.3 | 1969.1 ± 313.7 |
| eca-mir-146a-p3 | ACCTGTGAAGTTCAGTTCT | 3.1 ± 1.2 | 11.4 ± 8.5 |
| bta-miR-146b | TGAGAACTGAATTCCATAGGCTGT | 21.7 ± 8.6 | 7.7 ± 2.9 |
| hsa-miR-147b-5p_1ss9TC | TGGAAACACTTCTGCACAAACT | 1.4 ± 1.0 | --- |
| bta-miR-147_R-4 | GTGTGCGGAAATGCTTCT | 7.9 ± 2.3 | 1.5 ± 0.8 |
| hsa-miR-148a-5p | AAAGTTCTGAGACACTCCGACT | 1.9 ± 0.8 | 2.7 ± 1.7 |
| bta-miR-148a | TCAGTGCACTACAGAACTTTGT | 729.6 ± 176.6 | 936.6 ± 170.8 |
| hsa-miR-148b-5p_L+1 | GAAGTTCTGTTATACACTCAGGC | 2.9 ± 1.7 | 2.6 ± 1.8 |
| bta-miR-148b_R-2 | TCAGTGCATCACAGAACTTT | 148.7 ± 25.8 | 94.6 ± 15.0 |
| bta-miR-149-5p_R-3 | TCTGGCTCCGTGTCTTCACT | 6.8 ± 2.5 | 32.8 ± 9.4 |
| bta-miR-150_R-2 | TCTCCCAACCCTTGTACCAGT | 260.0 ± 123.9 | 194.4 ± 30.7 |
| bta-miR-151-5p | TCGAGGAGCTCACAGTCTAGT | 460.4 ± 92.6 | 415.6 ± 75.2 |
| bta-miR-151-3p | CTAGACTGAAGCTCCTTGAGG | 93.3 ± 14.5 | 99.8 ± 15.5 |
| bta-miR-152_R-1 | TCAGTGCATGACAGAACTTGG | 84.6 ± 21.8 | 125.8 ± 27.6 |
| bta-miR-153 | TTGCATAGTCACAAAAGTGATC | --- | 1.1 ± 0.7 |
| bta-miR-154c | AGATATTGCACGGTTGATCTCT | 0.4 ± 0.4 | --- |
| bta-miR-155 | TTAATGCTAATCGTGATAGGGGT | 12.9 ± 4.0 | 12.8 ± 2.4 |
| hsa-miR-181a-5p | AACATTCAACGCTGTCGGTGAGT | 386.5 ± 67.0 | 253.9 ± 34.2 |
| hsa-miR-181a-3p_1ss20AG | ACCATCGACCGTTGATTGTGCC | 2.7 ± 1.0 | 4.0 ± 1.0 |
| bta-miR-181b_R-6 | AACATTCATTGCTGTCGG | 48.1 ± 8.2 | 32.9 ± 5.3 |
| bta-miR-181d | AACATTCATTGTTGTCGGTGGGT | 19.5 ± 7.1 | 11.4 ± 5.1 |
| bta-miR-181c_R-2 | AACATTCAACCTGTCGGTGAGT | 87.4 ± 20.4 | 41.4 ± 17.4 |
| hsa-miR-181c-3p_L-1R-1 | ACCATCGACCGTTGAGTGGA | 0.3 ± 0.3 | 0.6 ± 0.6 |
| bta-miR-182_R-2 | TTTGGCAATGGTAGAACTCACA | 6.0 ± 3.0 | 20.4 ± 8.8 |
| bta-miR-183_R-1 | TATGGCACTGGTAGAATTCACT | --- | 2.5 ± 1.8 |
| bta-miR-184_1ss22TC | TGGACGGAGAACTGATAAGGGC | 2.3 ± 2.0 | 6.2 ± 6.2 |
| hsa-miR-185-5p | TGGAGAGAAAGGCAGTTCCTGA | 629.1 ± 158.5 | 369.2 ± 59.8 |
| hsa-miR-185-3p_R-2 | AGGGGCTGGCTTTCCTCTGG | 0.5 ± 0.5 | --- |
| bta-miR-186 | CAAAGAATTCTCCTTTTGGGCT | 1752.9 ± 354.7 | 730.7 ± 146.3 |
| bta-miR-188 | CATCCCTTGCATGGTGGAGGGT | 1.3 ± 0.7 | 0.9 ± 0.8 |
| bta-miR-190b_R-1 | TGATATGTTTGATATTGGGT | 9.1 ± 3.3 | 0.5 ± 0.5 |
| bta-miR-191_R-1 | CAACGGAATCCCAAAAGCAGCT | 17119.0 ± 1520.0 | 13057.8 ± 1089.1 |
| bta-miR-192_R-2 | CTGACCTATGAATTGACAGCC | 1366.7 ± 530.0 | 2149.2 ± 631.5 |
| hsa-miR-192-3p_R-1 | CTGCCAATTCCATAGGTCACA | 0.8 ± 0.8 | 0.2 ± 0.2 |
| bta-miR-193a-5p_R-2 | TGGGTCTTTGCGGGCGAGAT | 19.9 ± 6.4 | 25.6 ± 6.6 |
| bta-miR-193a-3p_R-4 | AACTGGCCTACAAAGTCC | 25.2 ± 15.3 | 16.6 ± 6.8 |
| hsa-miR-193b-5p_R-4 | CGGGGTTTTGAGGGCGAG | --- | 0.3 ± 0.3 |
| bta-miR-193b_R-2 | AACTGGCCCACAAAGTCCCGCT | 128.1 ± 55.3 | 99.0 ± 31.6 |
| bta-miR-194_R-1 | TGTAACAGCAACTCCATGTGG | 21.4 ± 9.6 | 30.3 ± 13.9 |
| mmu-miR-194-1-3p_R-1_1ss10CA | CCAGTGGAGATGCTGTTACTT | --- | 0.5 ± 0.5 |
| bta-miR-195 | TAGCAGCACAGAAATATTGGCA | 27.2 ± 5.3 | 26.3 ± 5.3 |
| bta-miR-196b_R-2 | TAGGTAGTTTCCTGTTGTTGG | 0.5 ± 0.5 | --- |
| hsa-miR-197-5p_R-2 | CGGGTAGAGAGGGCAGTGGGA | 0.3 ± 0.3 | --- |
| bta-miR-197 | TTCACCACCTTCTCCACCCAGC | 690.4 ± 165.4 | 206.3 ± 56.5 |
| bta-miR-199b_R-2 | CCCAGTGTTTAGACTATCTGT | 19.8 ± 4.4 | 36.4 ± 10.2 |
| bta-miR-199c_L-1R+2 | ACAGTAGTCTGCACATTGGTT | 32.9 ± 6.9 | 164.9 ± 31.6 |
| bta-miR-199a-5p_R+1 | CCCAGTGTTCAGACTACCTGTTC | 28.1 ± 4.8 | 98.7 ± 7.4 |
| bta-miR-200c | TAATACTGCCGGGTAATGATGGA | 1.1 ± 0.8 | 1.1 ± 0.8 |
| mmu-miR-200b-5p_R-1 | CATCTTACTGGGCAGCATTGG | --- | 1.4 ± 0.8 |
| bta-miR-200b | TAATACTGCCTGGTAATGATG | 3.4 ± 1.0 | 10.1 ± 7.9 |
| hsa-miR-200a-5p | CATCTTACCGGACAGTGCTGGA | --- | 0.2 ± 0.2 |
| bta-miR-200a_R-2 | TAACACTGTCTGGTAACGATG | 16.7 ± 4.0 | 40.0 ± 24.8 |
| cfa-miR-202_R-3 | TTCCTATGCATATACTTCT | 0.8 ± 0.8 | 1.8 ± 0.9 |
| bta-miR-204 | TTCCCTTTGTCATCCTATGCCT | 0.4 ± 0.4 | 3.0 ± 1.4 |
| bta-miR-205_R-1 | TCCTTCATTCCACCGGAGTCT | 205.2 ± 41.7 | 278.0 ± 91.2 |
| bta-miR-206_R-2 | TGGAATGTAAGGAAGTGTGT | 0.6 ± 0.6 | 0.8 ± 0.5 |
| bta-miR-208b_R-2 | ATAAGACGAACAAAAGGTTT | 8.0 ± 7.3 | 4.0 ± 2.7 |
| bta-miR-208a_R-1 | ATAAGACGAGCAAAAAGCTTG | 35.2 ± 13.5 | --- |
| hsa-miR-210-5p_R-1_1ss5CA | AGCCACTGCCCACCGCACACT | 0.3 ± 0.3 | --- |
| bta-miR-210_L-1R-2 | CTGTGCGTGTGACAGCGGCT | 40.7 ± 14.5 | 17.6 ± 7.2 |
| bta-miR-211_R+1 | TTCCCTTTGTCATCCTTTGCCT | 60.4 ± 28.1 | 42.0 ± 23.2 |
| hsa-miR-214-5p_1ss22CA | TGCCTGTCTACACTTGCTGTGA | --- | 0.2 ± 0.2 |
| bta-miR-214_L+1R-3 | TACAGCAGGCACAGACAGGC | 110.6 ± 29.5 | 145.2 ± 26.4 |
| bta-miR-215_R-1 | ATGACCTATGAATTGACAGAC | 1144.8 ± 673.1 | 783.3 ± 272.5 |
| hsa-miR-215-3p_R-1_1ss13TG | TCTGTCATTTCTGTAGGCCAAT | --- | 3.1 ± 2.6 |
| bta-miR-216a | TAATCTCAGCTGGCAACTGTGA | --- | 1.7 ± 0.8 |
| bta-miR-218_R-1 | TTGTGCTTGATCTAACCATGT | --- | 0.3 ± 0.3 |
| hsa-miR-219a-5p | TGATTGTCCAAACGCAATTCT | 5.1 ± 3.2 | 0.8 ± 0.8 |
| hsa-miR-221-5p_R-1 | ACCTGGCATACAATGTAGATT | 0.6 ± 0.4 | 17.4 ± 5.4 |
| bta-miR-221_R-1 | AGCTACATTGTCTGCTGGGTT | 677.4 ± 153.4 | 859.2 ± 284.3 |
| bta-miR-222 | AGCTACATCTGGCTACTGGGT | 63.7 ± 17.4 | 100.2 ± 30.2 |
| hsa-miR-223-5p_R-1 | CGTGTATTTGACAAGCTGAGT | 94.1 ± 31.8 | 100.5 ± 18.1 |
| bta-miR-223 | TGTCAGTTTGTCAAATACCCCA | 2501.9 ± 561.9 | 2480.2 ± 784.0 |
| bta-miR-301a_R-2 | CAGTGCAATAGTATTGTCAAAGC | 4.9 ± 1.5 | 5.0 ± 2.5 |
| bta-miR-301b_R-2 | CAGTGCAATGATATTGTCAAAGC | 0.1 ± 0.1 | --- |
| bta-miR-320a | AAAAGCTGGGTTGAGAGGGCGA | 2776.2 ± 708.8 | 1648.0 ± 388.0 |
| bta-miR-323_L-1_1ss6TA | CACAATACACGGTCGACCTCT | --- | 0.2 ± 0.2 |
| bta-miR-324_R-3 | CGCATCCCCTAGGGCATTGG | 4.3 ± 1.8 | 2.9 ± 2.0 |
| hsa-miR-324-3p_L-3R+1 | ACTGCCCCAGGTGCTGCTGGT | 3.5 ± 1.6 | 2.2 ± 1.5 |
| bta-miR-326_R-2 | CCTCTGGGCCCTTCCTCC | 26.4 ± 7.4 | 11.9 ± 4.1 |
| bta-miR-328 | CTGGCCCTCTCTGCCCTTCCGT | 47.9 ± 9.4 | 12.2 ± 2.5 |
| hsa-miR-330-5p_R-2 | TCTCTGGGCCTGTGTCTTAG | 1.4 ± 0.9 | 0.9 ± 0.7 |
| bta-miR-330 | GCAAAGCACACGGCCTGCAGAGA | 21.3 ± 8.0 | 18.6 ± 7.4 |
| bta-miR-331-5p_R+3 | TCTAGGTATGGTCCCAGGGAT | 69.8 ± 20.7 | 43.7 ± 9.6 |
| bta-miR-331-3p | GCCCCTGGGCCTATCCTAGAA | 0.6 ± 0.4 | 9.0 ± 3.0 |
| bta-miR-335_R-2 | TCAAGAGCAATAACGAAAAAT | 91.0 ± 24.7 | 93.8 ± 22.7 |
| hsa-miR-335-3p_R-2 | TTTTTCATTATTGCTCCTGA | 0.2 ± 0.2 | --- |
| hsa-miR-338-5p_R-1 | AACAATATCCTGGTGCTGAGT | 25.2 ± 6.3 | 32.5 ± 7.9 |
| bta-miR-338 | TCCAGCATCAGTGATTTTGTTGA | 23.2 ± 7.3 | 9.1 ± 3.7 |
| ssc-miR-339_R-2 | TCCCTGTCCTCCAGGAGCT | 377.3 ± 105.7 | 195.7 ± 36.3 |
| hsa-miR-340-5p | TTATAAAGCAATGAGACTGATT | 172.0 ± 38.4 | 254.1 ± 100.8 |
| bta-miR-340_R-1 | TCCGTCTCAGTTACTTTATAGC | 0.7 ± 0.5 | 1.6 ± 0.7 |
| hsa-miR-342-3p | TCTCACACAGAAATCGCACCCGT | 1040.6 ± 155.9 | 583.2 ± 103.2 |
| bta-miR-345-5p_R+1 | GCTGACTCCTAGTCCAGTGCTC | 1.5 ± 0.7 | 6.7 ± 2.5 |
| bta-miR-345-3p_L+1_1ss13GA | CCCTGAACTAGGAGTCTGGAG | 0.9 ± 0.5 | 2.9 ± 1.6 |
| mmu-miR-350-3p_R-1 | TTCACAAAGCCCATACACTTT | 8.1 ± 3.1 | 1.8 ± 1.2 |
| bta-miR-361_R-2 | TTATCAGAATCTCCAGGGGT | 157.5 ± 26.0 | 150.0 ± 9.5 |
| hsa-miR-361-3p_R-1 | TCCCCCAGGTGTGATTCTGATT | 11.5 ± 3.2 | 7.2 ± 4.2 |
| bta-miR-362-5p_R-2 | AATCCTTGGAACCTAGGTGTGA | 0.4 ± 0.4 | 0.9 ± 0.6 |
| bta-miR-362-3p_R-1 | AACACACCTATTCAAGGATT | 1.8 ± 0.9 | 4.1 ± 2.3 |
| bta-miR-363_L+1R-1_1ss21CT | AATTGCACGGTATCCATCTGT | 17.4 ± 4.3 | 11.7 ± 3.6 |
| bta-miR-365-3p_R-3 | TAATGCCCCTAAAAATCCT | 32.8 ± 10.8 | 30.3 ± 11.0 |
| bta-miR-369-3p_R-1 | AATAATACATGGTTGATCTT | --- | 0.2 ± 0.2 |
| bta-miR-370_R-4 | GCCTGCTGGGGTGGAACC | --- | 0.2 ± 0.2 |
| bta-miR-374a_R-1 | TTATAATACAACCTGATAAGT | 174.1 ± 34.0 | 231.3 ± 89.3 |
| hsa-miR-374a-3p_1ss9AG | CTTATCAGGTTGTATTGTAATT | 1.4 ± 0.8 | 0.3 ± 0.3 |
| bta-miR-374b_R-1 | ATATAATACAACCTGCTAAGT | 18.1 ± 4.6 | 25.6 ± 12.3 |
| bta-miR-375_L-1R-3 | TTTGTTCGTTCGGCTCGCG | 38.7 ± 9.7 | 54.5 ± 15.2 |
| bta-miR-377_R-1 | ATCACACAAAGGCAACTTTTG | 0.5 ± 0.5 | --- |
| hsa-miR-378a-5p | CTCCTGACTCCAGGTCCTGTGT | 0.4 ± 0.3 | 0.5 ± 0.5 |
| bta-miR-378 | ACTGGACTTGGAGTCAGAAGGC | 253.6 ± 89.2 | 142.1 ± 44.9 |
| bta-miR-381_R-2 | TATACAAGGGCAAGCTCTCT | 2.0 ± 1.5 | 0.8 ± 0.6 |
| hsa-miR-409-3p | GAATGTTGCTCGGTGAACCCCT | --- | 0.4 ± 0.4 |
| bta-miR-410_R-1 | AATATAACACAGATGGCCTG | --- | 0.3 ± 0.3 |
| bta-miR-421_R-1 | ATCAACAGACATTAATTGGGCG | 4.8 ± 2.0 | 3.2 ± 2.2 |
| bta-miR-423-5p_R-2 | TGAGGGGCAGAGAGCGAGACT | 2504.0 ± 827.5 | 1291.9 ± 201.0 |
| bta-miR-423-3p_L-1 | AGCTCGGTCTGAGGCCCCTCAGT | 926.3 ± 298.2 | 611.9 ± 89.9 |
| bta-miR-424-5p | CAGCAGCAATTCATGTTTTGA | 132.3 ± 36.5 | 120.9 ± 93.8 |
| bta-miR-424-3p_R-2 | CAAAACGTGAGGCGCTGCT | 3.6 ± 1.4 | 8.6 ± 2.8 |
| bta-miR-425-5p_L+1R-1 | AATGACACGATCACTCCCGTTG | 686.5 ± 116.6 | 319.6 ± 78.3 |
| bta-mir-425-p3_1ss18CT | ATCGGGAATGTCGTGTCTGC | 2.3 ± 1.0 | 1.9 ± 1.0 |
| bta-miR-429_R-1 | TAATACTGTCTGGTAATGCCG | 4.2 ± 1.8 | 6.4 ± 3.2 |
| bta-miR-432_R-3 | TCTTGGAGTAGGTCATTGGG | --- | 0.7 ± 0.7 |
| bta-miR-450a | TTTTGCGATGTGTTCCTAATAT | 8.2 ± 2.2 | 17.9 ± 8.4 |
| bta-miR-450b_R-1 | TTTTGCAATATGTTCCTGAAT | 91.2 ± 20.7 | 104.1 ± 65.0 |
| bta-miR-451_R-2 | AAACCGTTACCATTACTGAGT | 217814.9 ± 59526.9 | 39966.1 ± 11020.9 |
| bta-mir-451-p3 | TTTAGTAATGGTAACGGTTCT | 8.3 ± 2.4 | 5.5 ± 1.8 |
| mmu-mir-451b-p5_1ss18AG | GAACCGTTACCATTACTGA | 1.6 ± 0.7 | 0.1 ± 0.1 |
| hsa-miR-454-5p_R-1_1ss9AG | ACCCTATCGATATTGTCTCTG | --- | 0.2 ± 0.2 |
| bta-miR-454 | TAGTGCAATATTGCTTATAGGGT | 0.5 ± 0.5 | 1.5 ± 1.2 |
| bta-miR-455-5p_R+1 | TATGTGCCTTTGGACTACATCG | --- | 1.0 ± 0.6 |
| bta-miR-483_R+2 | TCACTCCTCTCCTCCCGTCTTCT | 1.3 ± 0.9 | 0.2 ± 0.2 |
| bta-miR-484_R-1 | TCAGGCTCAGTCCCCTCCCGA | 771.1 ± 271.0 | 484.5 ± 150.5 |
| hsa-miR-486-3p_R-1 | CGGGGCAGCTCAGTACAGGA | 94.9 ± 51.7 | 49.6 ± 17.1 |
| bta-miR-486_R+1 | TCCTGTACTGAGCTGCCCCGAGT | 34277.2 ± 8895.5 | 14237.0 ± 2635.6 |
| bta-miR-491 | AGTGGGGAACCCTTCCATGAGG | 0.3 ± 0.3 | 0.2 ± 0.2 |
| bta-miR-497_R-1 | CAGCAGCACACTGTGGTTTGT | 16.5 ± 4.9 | 15.1 ± 4.5 |
| bta-miR-499 | TTAAGACTTGCAGTGATGTTT | 8.2 ± 2.6 | 29.4 ± 9.5 |
| hsa-miR-501-5p_1ss1AT | TATCCTTTGTCCCTGGGTGAGA | --- | 0.1 ± 0.1 |
| bta-miR-502a_R-2 | AATGCACCTGGGCAAGGATT | 31.9 ± 8.5 | 56.1 ± 17.7 |
| eca-mir-503-p3_1ss23GA | GAGTATTGTTCCTGCTGCCCGGA | 0.4 ± 0.3 | 0.2 ± 0.2 |
| bta-miR-504_R-4 | AGACCCTGGTCTGCACTC | --- | 0.6 ± 0.6 |
| hsa-miR-505-5p_R+2 | GGGAGCCAGGAAGTATTGATGTTT | 0.6 ± 0.6 | --- |
| bta-miR-505_L-2R+2 | TCAACACTTGCTGGTTTCCTCT | 86.7 ± 24.3 | 105.8 ± 34.7 |
| bta-miR-532 | CATGCCTTGAGTGTAGGACCGT | 21.1 ± 6.7 | 33.0 ± 9.4 |
| hsa-miR-532-3p_R-4 | CCTCCCACACCCAAGGCT | 4.5 ± 2.0 | 5.7 ± 1.8 |
| bta-miR-542-5p_R+1 | TCGGGGATCATCATGTCACGAGA | 2.5 ± 1.4 | 0.6 ± 0.6 |
| hsa-miR-542-3p_R-2 | TGTGACAGATTGATAACTGA | 1.5 ± 0.9 | 7.6 ± 4.7 |
| bta-miR-543_R-1 | AAACATTCGCGGTGCACTTCT | --- | 0.2 ± 0.2 |
| bta-miR-545-3p_R-1 | ATCAACAAACATTTATTGTGT | 0.1 ± 0.1 | 0.8 ± 0.7 |
| hsa-miR-574-5p_R-5 | TGAGTGTGTGTGTGTGAG | 0.2 ± 0.2 | 0.6 ± 0.6 |
| hsa-miR-574-3p | CACGCTCATGCACACACCCACA | 59.1 ± 13.9 | 88.2 ± 24.3 |
| bta-miR-582_R-3_1ss17AG | TTACAGTTGTTCAACCGGTT | 1.5 ± 0.6 | 1.2 ± 0.8 |
| hsa-miR-582-3p_R-1_1ss5TC | TAACCGGTTGAACAACTGAAC | --- | 1.0 ± 0.8 |
| hsa-miR-590-3p | TAATTTTATGTATAAGCTAGT | 0.5 ± 0.4 | --- |
| bta-miR-592_L-1 | TTGTGTCAATATGCGATGATGT | 0.3 ± 0.3 | --- |
| bta-mir-599-p5 | TTTGATAAGCTGACATGGGACA | 0.2 ± 0.2 | --- |
| hsa-miR-615-3p_R-4 | TCCGAGCCTGGGTCTCCC | 5.7 ± 4.0 | 6.9 ± 2.4 |
| bta-miR-628 | ATGCTGACATATTTACTAGAGG | 0.2 ± 0.2 | 3.0 ± 1.8 |
| ptr-miR-628_R+2 | TCTAGTAAGAGTGGCAGTCGAAG | 79.4 ± 14.3 | 117.3 ± 19.9 |
| bta-miR-652_R-1 | AATGGCGCCACTAGGGTTGT | 59.1 ± 12.2 | 65.9 ± 12.6 |
| hsa-miR-653-3p_R-1 | TTCACTGGAGTTTGTTTCAAT |  | 0.2 ± 0.2 |
| bta-miR-656 | AATATTATACAGTCAACCTCT | 0.9 ± 0.7 | 0.2 ± 0.2 |
| hsa-miR-660-5p | TACCCATTGCATATCGGAGTTG | 47.2 ± 14.5 | 45.0 ± 18.2 |
| hsa-miR-660-3p_R-2 | ACCTCCTGTGTGCATGGAT | --- | 0.2 ± 0.2 |
| hsa-miR-671-3p_R-3 | TCCGGTTCTCAGGGCTCC | --- | 0.8 ± 0.8 |
| eca-miR-675_R-4 | TGGCGCGGAGAGGGCCCAC | --- | 1.6 ± 1.2 |
| eca-miR-676_R-1 | CCGTCCTAAGGTTGTTGAGT | 3.1 ± 2.0 | 31.1 ± 9.1 |
| bta-miR-708_R-3 | AAGGAGCTTACAATCTAGCT | --- | 0.2 ± 0.2 |
| bta-miR-744_R-1 | TGCGGGGCTAGGGCTAACAGC | 13.4 ± 4.2 | 13.6 ± 3.2 |
| hsa-miR-766-3p | ACTCCAGCCCCACAGCCTCAGC | 1.3 ± 0.9 | 1.0 ± 0.7 |
| hsa-miR-802_L+1R-2 | TCAGTAACAAAGATTCATCCTT | 64.9 ± 43.7 | 36.0 ± 21.5 |
| bta-miR-873_L+1R-3 | TGCAGGAACTTGTGAGTCT | 32.8 ± 13.4 | 4.3 ± 2.9 |
| bta-miR-874_R+1 | CTGCCCTGGCCCGAGGGACCGAC | 0.8 ± 0.5 | 12.5 ± 2.9 |
| bta-miR-875_R-2 | TATACCTCAGTTTTATCAGG | 0.2 ± 0.2 | --- |
| hsa-miR-877-3p_R+1 | TCCTCTTCTCCCTCCTCCCAGT | --- | 2.0 ± 1.6 |
| bta-miR-885 | TCCATTACACTACCCTGCCTCT | 35.1 ± 29.6 | 30.2 ± 16.6 |
| eca-miR-1180_R-2 | TTTCCGGCTCGAGTGGGTGT | 0.7 ± 0.5 | 0.2 ± 0.2 |
| bta-miR-1224 | GTGAGGACTCGGGAGGTGGAG | 2.1 ± 1.6 | 0.2 ± 0.2 |
| bta-miR-1247-5p | ACCCGTCCCGTGCGTCCCCGGA | --- | 0.3 ± 0.3 |
| eca-mir-1248-p5 | AAATTGCAGAAACTAGGAT | 0.3 ± 0.3 | --- |
| bta-miR-1249 | ACGCCCTTCCCCCCCTTCTTCA | 28.7 ± 6.0 | 11.1 ± 5.6 |
| eca-miR-1271a_R-2 | CTTGGCACCTCGTAAGCACT | 130.2 ± 24.0 | 128.8 ± 27.7 |
| eca-mir-1271a-p3 | AGTGCCTGCTGTGTGCCAGG | 5.6 ± 2.9 | 0.6 ± 0.4 |
| bta-miR-1277_R-1 | TACGTAGATATATATGTATTT | 0.3 ± 0.3 | --- |
| ssc-mir-1285-p5_1ss12TC | AATAGCCACTGCACTCCAGCC | 4.3 ± 3.6 | 0.2 ± 0.2 |
| ssc-mir-1285-p3 | ATAGCGAGACCCCGTCTC | 412.0 ± 176.4 | 606.0 ± 296.7 |
| hsa-miR-1291_L+1 | GTGGCCCTGACTGAAGACCAGCAGT | 115.2 ± 67.8 | 120.4 ± 49.5 |
| bta-miR-1296_R-2 | TTAGGGCCCTGGCTCCATCT | 1.0 ± 0.7 | 0.3 ± 0.3 |
| hsa-miR-1301-3p_R-4 | TTGCAGCTGCCTGGGAGTGA | 0.7 ± 0.5 | 0.3 ± 0.3 |
| bta-miR-1306_R-1 | CCACCTCCCCTGCAAACGTC | 5.7 ± 2.0 | 8.7 ± 4.7 |
| hsa-miR-1306-3p_R+1 | ACGTTGGCTCTGGTGGTGA | 0.6 ± 0.6 | --- |
| hsa-miR-1307-5p | TCGACCGGACCTCGACCGGCT | 116.9 ± 25.6 | 65.3 ± 18.9 |
| bta-miR-1307_R-2 | ACTCGGCGTGGCGTCGGTCG | 22.3 ± 6.3 | 26.4 ± 8.3 |
| ggo-miR-1343 | CTCCTGGGGCCCGCACTC | --- | 2.0 ± 2.0 |
| bta-miR-1343-3p_R+1 | CTCCTGGGGCCCGCACTCTCG | 29.9 ± 13.8 | 62.4 ± 46.1 |
| eca-miR-1379_L-1R-3 | CACGGGCTGCAGCGCCGACG | 7.7 ± 2.4 | 21.7 ± 7.8 |
| eca-miR-1388_R-3_1ss12AG | AGGACTGTCCAGCCTGAGAAT | 10.7 ± 3.0 | 41.2 ± 12.5 |
| eca-mir-1543-p5_1ss21GA | TTTGCACCTCTGAGAGTGGAA | 0.6 ± 0.6 | 0.6 ± 0.4 |
| ssc-miR-1839-5p_R-2 | AAGGTAGATAGAACAGGTCT | 2.2 ± 2.2 | 5.6 ± 2.5 |
| bta-miR-1842_L+1R-2_1ss10TC | TTGGCTCTGCGAGGTCGGCT | 67.5 ± 11.1 | 43.0 ± 8.3 |
| bta-mir-1842-p3 | AGCAGGCCTGTCAGGGCGTT | 4.4 ± 2.0 | 1.0 ± 1.0 |
| mmu-mir-1983-p5 | GCTCCAGTGGCGCAATCGG | 6.9 ± 2.5 | 21.6 ± 12.2 |
| mmu-mir-1983-p3 | TTCGAGCCTCACCTGGAGC | 0.9 ± 0.7 | --- |
| bta-miR-2483-5p_R-2 | CGTCAACCATCCAGCTGTTT | 124.4 ± 32.0 | 7.7 ± 4.3 |
| bta-miR-2483-3p | AAACATCTGGTTGGTTGAGAGA | 1439.1 ± 401.9 | 112.3 ± 29.2 |
| bta-miR-3065_R-1 | TCAACAAAATCACTGATGCTGG | 2.3 ± 0.9 | 0.2 ± 0.2 |
| bta-miR-3120 | CACAGCAAGTGTAGACAGGCA | 1.9 ± 1.4 | --- |
| hsa-miR-3200-5p | AATCTGAGAAGGCGCACAAGGT | 1.0 ± 0.7 | --- |
| hsa-miR-3200-3p_R-1 | CACCTTGCGCTACTCAGGTCT | 7.9 ± 2.5 | 0.2 ± 0.2 |
| bta-miR-3431_R-2_1ss9AT | CCTCAGTCTGCCTTGTGGAT | --- | 0.2 ± 0.2 |
| bta-miR-6119-5p_R-2 | AGAGGTAAAAAATTGATTTGA | 188.2 ± 41.1 | 104.8 ± 25.9 |
| bta-miR-6119-3p_L-1R-1 | CAAATCATTTTTTACTCTCCA | 9.1 ± 3.3 | 7.5 ± 4.6 |
| bta-mir-6517-p5_1ss4AC | CTCCGGGTCCGTGAGCTCCT | 0.2 ± 0.2 | --- |
| bta-miR-6529a | GAGAGATCAGAGGCGCAGAGT | 206.5 ± 58.9 | 106.1 ± 18.1 |
| hsa-miR-6529-3p_R-2_1ss15TC | CCTGTGCCTTTTACCTCTTT | 0.2 ± 0.2 | 0.4 ± 0.2 |
| eca-mir-8986a-p5 | GTCGAGGCTAGAGTCACGCTTGGGT | 3.3 ± 2.3 | 30.5 ± 14.4 |
| eca-mir-8986a-p3 | AGTCCTCGAAGAGTAACTGCTGACCT | --- | 0.2 ± 0.2 |
| eca-mir-9010-p3_1ss5GC | AGGTCCTGTGGCCCTGGC | --- | 0.6 ± 0.6 |
| eca-miR-9055_R-5 | AGAGAGCACCAAGGGAGC | 0.4 ± 0.3 | 4.8 ± 1.4 |
| efu-mir-9277-p3_1ss16AT | CCCCGCGCAGGTTCGTATCCTG | 5.3 ± 2.8 | 120.5 ± 63.8 |
| ssc-miR-9851-3p_R-4 | TGGCACCAGCACTGGCGG | 6.6 ± 4.0 | 40.3 ± 12.1 |
| mdo-miR-22-3p | AAGCTGCCAGTTGAAGAACTGC | 2.3 ± 1.3 | 0.3 ± 0.3 |
| mmu-miR-146a-5p_R+1 | TGAGAACTGAATTCCATGGGTTA | 50.3 ± 36.0 | 34.4 ± 13.1 |
| mdo-miR-150-5p_R-2 | TCTCCCAACCCTTGTACCAGA | 0.5 ± .5 | --- |
| mdo-miR-200a-3p_R+2 | TAACACTGTCTGGTAACGATGTTT | --- | 0.2 ± 0.2 |
| mmu-miR-215-5p_R+2_1ss12TA | ATGACCTATGAATTGACAGACAT | --- | 0.3 ± 0.3 |
| mmu-miR-300-3p_R-2 | TATGCAAGGGCAAGCTCTCT | --- | 0.9 ± 0.7 |
| hsa-miR-320d | AAAAGCTGGGTTGAGAGGA | 1.0 ± 0.6 | 2.6 ± 1.2 |
| hsa-miR-320b_R-1 | AAAAGCTGGGTTGAGAGGGCA | 4.4 ± 1.7 | 3.6 ± 2.1 |
| mmu-miR-326-3p | CCTCTGGGCCCTTCCTCCAGT | 35.7 ± 7.3 | 12.6 ± 2.8 |
| bta-miR-342_R-3 | TCTCACACAGAAATCGCACCCA | 47.4 ± 32.0 | --- |
| bta-miR-378_R+1 | ACTGGACTTGGAGTCAGAAGGCT | 20.6 ± 7.3 | 11.9 ± 4.0 |
| mmu-miR-486b-5p_R+2 | TCCTGTACTGAGCTGCCCCGAGGT | 37.6 ± 13.8 | 28.5 ± 6.7 |
| mmu-miR-664-3p_R-1_1ss13CT | TATTCATTTACTTCCCAGCCT | --- | 0.2 ± 0.2 |
| ppy-miR-1246_L+1R-1 | AAATGGGTTTTTGGAGCAG | --- | 4.0 ± 2.9 |
| bta-miR-1246_R-1 | AATGGATTTTTGGAGCAG | 42.3 ± 19.4 | 32.1 ± 24.5 |
| oan-miR-1329-5p_R+1_1ss20TA | TACAGTGATCAGGTTACGAAGGA | --- | 1.7 ± 1.1 |
| oan-miR-1386_L+1_1 | ACTCCTGGCTGGCTCGCCA | 58.0 ± 48.0 | 3.0 ± 1.6 |
| oan-miR-1386_L+1_3 | GCTCCTGGCTGGCTCGCCA | 58.0 ± 48.0 | 3.0 ± 1.6 |
| oan-miR-1388-3p_R-1 | ATCTCAGGTTCGTCAGCCCAT | 6.7 ± 2.4 | 5.7 ± 2.6 |
| bta-mir-2904-1-p3_1 | CGGCAGCGCCGCGGGAGCC | 0.2 ± 0.2 | 10.4 ± 6.1 |
| hsa-mir-3195-p5_1ss3CG | GGGCCCCGGCGGCCGGGG | 13.2 ± 9.3 |  |
| mmu-mir-6240-p5_1ss19GT | TCTGCCCAGTGCTCTGAATGTC | 376.3 ± 187.1 | 118.8 ± 47.0 |
| mmu-mir-6240-p5_1ss2TA | AAGTGATTTCTGCCCAGTG | --- | 0.1 ± 0.1 |
| efu-mir-9341-p3_1ss3TC_2 | ATCGCTTCTCGGCCTTTTGG | 7.0 ± 4.4 | 8.7 ± 4.5 |
| hsa-miR-10527-5p | AAAGCAAATGTTGGGTGAACGGC | 0.6 ± 0.5 | 0.6 ± 0.4 |
| oan-miR-145-5p_R+1_1ss19TA | GTCCAGTTTTCCCAGGAAAA | 0.8 ± 0.4 | 0.4 ± 0.3 |
| hsa-miR-151b_R+3 | TCGAGGAGCTCACAGTCTAGA | --- | 0.1 ± 0.1 |
| hsa-miR-203a-3p_L-1R-2 | TGAAATGTTTAGGACCACT | 13.5 ± 5.3 | 35.5 ± 8.1 |
| hsa-miR-203a-3p_R-3 | GTGAAATGTTTAGGACCAC | 14.1 ± 5.1 | 19.2 ± 8.2 |
| hsa-miR-203a-3p_R-4 | GTGAAATGTTTAGGACCA | 38.5 ± 13.5 | 72.4 ± 18.8 |
| oan-miR-223-3p_R-1 | TGTCAGTTTGTCAAATACCCT | 1.6 ± 0.6 | 13.3 ± 6.6 |
| hsa-miR-320d_R+1 | AAAAGCTGGGTTGAGAGGAA | 0.5 ± 0.4 | 0.9 ± 0.6 |
| mmu-miR-326-3p_1ss20GT | CCTCTGGGCCCTTCCTCCATT | 0.7 ± 0.5 | --- |
| mmu-miR-326-3p_R+1 | CCTCTGGGCCCTTCCTCCAGTT | 5.4 ± 1.6 | 3.2 ± 1.7 |
| efu-mir-339-p5 | TCCCTGTCCTCCAGGAGCTCACC | --- | 0.1 ± 0.1 |
| ssc-miR-339_R+1 | TCCCTGTCCTCCAGGAGCTCAT | 0.6 ± 0.4 | --- |
| hsa-miR-378c_R-4 | ACTGGACTTGGAGTCAGAAGA | 0.1 ± 0.1 | --- |
| mmu-mir-709-p5_1ss6AT | GAGGCTGAGGCAGGAGGAT | 9.7 ± 9.7 | 0.2 ± 0.2 |
| bta-miR-1246_1ss8TG | AATGGATGTTTGGAGCAGG | --- | 0.7 ± 0.7 |
| bta-miR-1246_1ss1AC | CATGGATTTTTGGAGCAGG | --- | 0.9 ± 0.9 |
| bta-miR-1246_L+1 | AAATGGATTTTTGGAGCAGG | 819.1 ± 466.7 | 2180.1 ± 1765.2 |
| ppy-miR-1246_R-1 | AATGGGTTTTTGGAGCAG | --- | 0.3 ± 0.3 |
| bta-miR-1246_L-2R+1 | TGGATTTTTGGAGCAGGG | 51.4 ± 37.8 | 93.9 ± 63.3 |
| bta-miR-1246_1ss2AG | AGTGGATTTTTGGAGCAGG | 5.4 ± 4.7 | 0.8 ± 0.6 |
| bta-miR-1246_L+1R+1 | AAATGGATTTTTGGAGCAGGG | 987.8 ± 571.6 | 1737.9 ± 1319.2 |
| ppy-miR-1246 | AATGGGTTTTTGGAGCAGG | --- | 2.6 ± 1.9 |
| bta-miR-1246_1ss1AG | GATGGATTTTTGGAGCAGG | 0.6 ± 0.5 | --- |
| cgr-miR-1260_R+2_2 | ATCCCACCGCTGCCACCAGA | 0.1 ± 0.1 | --- |
| cgr-miR-1260_L+1 | AATCCCACCGCTGCCACCA | --- | 0.3 ± 0.3 |
| cgr-miR-1260 | ATCCCACCGCTGCCACCA | 14.2 ± 7.5 | 5.3 ± 4.6 |
| cgr-miR-1260_R+2_1 | ATCCCACCGCTGCCACCAAA | 0.1 ± 0.1 | --- |
| cfa-miR-1271_R+2 | CTTGGCACCTAGTAAGCACTCA | 2.2 ± 1.8 | 4.0 ± 2.1 |
| cgr-mir-1285-p3 | ACTGCACTCCAGCCTGGGC | 6.2 ± 4.5 | 2.5 ± 1.5 |
| cgr-miR-1285_R-6 | TGCACTCCAGCCTGGGCA | 1.5 ± 1.5 | --- |
| cgr-miR-1285_L+1R-7 | CTGCACTCCAGCCTGGGC | 4.7 ± 3.2 | 6.4 ± 3.7 |
| oan-mir-1383-p3_1ss19TG | TCACTGGTTTTTCCTTGGGC | --- | 0.4 ± 0.4 |
| oan-miR-1386_L+1R-1_2 | GCTCCTGGCTGGCTCGCC | 5.9 ± 4.8 | --- |
| oan-miR-1386_L+1R-1_1 | CCTCCTGGCTGGCTCGCC | 5.9 ± .8 | --- |
| oan-miR-1386_L+2R-1_1 | GGCTCCTGGCTGGCTCGCC | 27.4 ± 25.4 | 0.3 ± 0.3 |
| oan-miR-1386_L+1_2 | CCTCCTGGCTGGCTCGCCA | 58.0 ± 48.0 | 3.0 ± 1.6 |
| oan-miR-1386_L+2 | GGCTCCTGGCTGGCTCGCCA | 9.3 ± 9.1 | --- |
| oan-miR-1386_L+2R-1_2 | GTCTCCTGGCTGGCTCGCC | 27.4 ± 25.4 | 0.3 ± 0.3 |
| oan-mir-1386-p5_1ss9GC | GGCTCCTGCCTGGCTCGCC | 7.6 ± 7.6 | --- |
| oan-miR-1386_L+1R-1_3 | TCTCCTGGCTGGCTCGCC | 5.9 ± 4.8 | --- |
| oan-miR-1386_1ss7GC | CTCCTGCCTGGCTCGCCA | 2.4 ± 2.4 | 0.1 ± 0.1 |
| oan-mir-1386-p3_1ss2GC | TCGGCTCCTGGCTGGCTCGC | 0.4 ± 0.4 | --- |
| oan-miR-1386_L+4 | TCGGCTCCTGGCTGGCTCGCCA | 1.8 ± 1.3 | 1.3 ± 0.9 |
| hsa-miR-1973_R-1_1ss17AG | ACCGTGCAAAGGTAGCGT | 0.5 ± 0.5 | --- |
| mmu-miR-2137_L-3_1ss16AG | GGCGGGAGCCCCGGGGAG | --- | 0.8 ± 0.8 |
| mmu-miR-2137_L-2_1ss16AG | CGGCGGGAGCCCCGGGGAG | --- | 14.9 ± 14.6 |
| mmu-miR-2137_L-1_1ss16AG | CCGGCGGGAGCCCCGGGGAG | --- | 1.5 ± 1.5 |
| mmu-miR-2137_L-2R-1_1ss16AG | CGGCGGGAGCCCCGGGGA | 2.4 ± 2.4 | 9.2 ± 8.3 |
| bta-mir-2369-p3_1ss12TA | GGTTGTGGGTTATTGTTTC | 2292.9 ± 1378.8 | --- |
| bta-miR-2478_L+2R-1 | TCGTATCCCACTTCTGACACC | 0.4 ± 0.2 | 1.0 ± 1.0 |
| bta-mir-2478-p3_1ss9TC | ATCCCACTCCTGACACCAT | --- | 0.7 ± 0.7 |
| bta-miR-2478_L+2 | TCGTATCCCACTTCTGACACCA | 2.1 ± 1.8 | 0.6 ± 0.6 |
| bta-mir-2887-2-p5 | GACCGGGGTCCGGTGCGGAGAGCCC | 10.2 ± 3.7 | 39.2 ± 18.5 |
| bta-mir-2887-2-p3_1ss1CT | TGGGACACGGGGCGCGGCC | 6.5 ± 3.9 | 83.5 ± 64.7 |
| bta-miR-2887_R+6 | CGGGACCGGGGTCCGGTGCGGAGAGC | 5.5 ± 5.5 | 1.3 ± 1.0 |
| bta-miR-2904_R-1 | GGGAGCCTCGGTTGGCCT | 1.2 ± 1.2 | 3.9 ± 2.4 |
| bta-miR-2904_L+1R-2 | CGGGAGCCTCGGTTGGCC | 1.2 ± 1.2 | 7.7 ± 4.9 |
| bta-mir-2904-1-p3_2 | GCCTCGGTTGGCCTCGGA | 0.2 ± 0.2 | 10.4 ± 6.1 |
| bta-mir-2904-1-p5 | GGCAGCGCCGCGGGAGCC | 1.5 ± 1.2 | 9.3 ± 8.0 |
| hsa-miR-3135b_R-2_1ss7AT | GGCTGGTGCGAGTGCAGTGG | 1.5 ± 1.0 | --- |
| mmu-miR-3535_L-1_1ss20CT | GGATATGATGACTGATTATCTGAGA | --- | 1.7 ± 1.1 |
| mmu-mir-3535-p3 | TTCCTCTAGATAGTCAAGTTCTGATC | 0.3 ± 0.3 | 3.4 ± 3.4 |
| mmu-miR-3968_L-2_1ss14AT | AATCCCACTCCTGACACCA | 0.6 ± 0.5 | 1.1 ± 1.0 |
| mmu-miR-3968_1ss14AT | CGAATCCCACTCCTGACACCA | 0.5 ± 0.5 | --- |
| mmu-miR-3968_L-3_1ss14AT | ATCCCACTCCTGACACCA | 2.4 ± 1.1 | 5.5 ± 3.2 |
| bta-miR-4286_R+2 | ACCCCACTCCTGGTACCAA | --- | 0.1 ± 0.1 |
| bta-miR-4286_R+1 | ACCCCACTCCTGGTACCA | 0.1 ± 0.1 | 0.1 ± 0.1 |
| hsa-miR-4448_R-2_1ss6CG | GGCTCGTTGGTCTAGGGG | --- | 2.6 ± 1.3 |
| hsa-miR-4454_L-2 | ATCCGAGTCACGGCACCA | 10.7 ± 6.1 | 9.8 ± 3.1 |
| hsa-miR-4492_L+1 | CGGGGCTGGGCGCGCGCC | --- | 0.4 ± 0.4 |
| mmu-miR-5100_R-2 | TCGAATCCCAGCGGTGCCT | 8.8 ± 3.7 | 9.5 ± 4.7 |
| mmu-miR-5100_1ss21TC | TCGAATCCCAGCGGTGCCTCC | 13.5 ± 9.8 | 4.5 ± 2.9 |
| mmu-miR-5100_R-2_1ss15TG | TCGAATCCCAGCGGGGCCT | 0.1 ± 0.1 | --- |
| mmu-miR-5100_L-1R-1_1ss15TG | CGAATCCCAGCGGGGCCTC | --- | 0.3 ± 0.3 |
| efu-miR-9226_L-1R-3_1ss4AG | CAGGTCCCTGTTCGGGCG | 0.3 ± 0.3 | --- |
| mmu-mir-5108-p3_1ss2TG | GGGGTAGAGCACTGGATGG | 6.6 ± 5.2 | 1.4 ± 0.7 |
| mmu-mir-5119-p5_1ss1GC | CTCATCTCATCCTGGGGCT | 5.5 ± 4.0 | 0.6 ± 0.4 |
| mmu-mir-5119-p3 | TCATCTCATCCTGGGGCT | 5.9 ± 3.4 | 1.2 ± 0.7 |
| mmu-mir-5119-p5_1ss6TA | TCATCACATCCTGGGGCTG | 1.4 ± 1.4 | --- |
| mmu-mir-5119-p5_1ss6TG | TCATCGCATCCTGGGGCT | 10.0 ± 3.3 | 4.4 ± 2.1 |
| mmu-miR-5119_R-1_1ss5TA | CATCACATCCTGGGGCTG | 0.5 ± 0.4 | 0.2 ± 0.1 |
| mmu-miR-5119_1ss19GT | CATCTCATCCTGGGGCTGT | 3.2 ± 2.1 | 0.3 ± 0.3 |
| mmu-miR-5119_L+1R-1 | TCATCTCATCCTGGGGCTG | 0.1 ± 0.1 | --- |
| mmu-miR-5119_L+1_1ss6TG | TCATCGCATCCTGGGGCTGG | 2.1 ± 0.9 | 0.2 ± 0.1 |
| mmu-miR-5124a_L-1_1ss5CA | GTCAAGTGACTAAGAGCAT | 7.1 ± 4.6 | 4.4 ± 1.2 |
| mmu-miR-5124a_L-1R-1_1ss5CA | GTCAAGTGACTAAGAGCA | 66.8 ± 42.2 | 28.2 ± 8.2 |
| mmu-miR-5126_L+1R-5 | GGCGGGCGGGGCCGGGGG | --- | 0.7 ± 0.7 |
| mmu-mir-6236-p5_5 | GAATCAACTAGCCCTGAAA | 1.8 ± 1.8 | 0.1 ± 0.1 |
| mmu-mir-6236-p3_1ss21GA | CCTGAAAATGGATGGCGCTGAAGC | 63.0 ± 48.7 | 34.5 ± 13.1 |
| mmu-mir-6236-p5_1ss3CG | ATGAACTAGCCCTGAAAATGGA | 11.8 ± 8.0 | 31.4 ± 30.9 |
| mmu-mir-6236-p3_2 | ACTAGCCCTGAAAATGGATGGCGCT | 4.0 ± 3.3 | --- |
| mmu-mir-6236-p5_4 | CAACTCACCTGCCGAATC | 1.8 ± 1.8 | 0.1 ± 0.1 |
| mmu-mir-6236-p3_1 | ACAACTCACCTGCCGAAT | 4.0 ± 3.3 | --- |
| mmu-mir-6236-p3_1ss12CG | ACCTGCCGAATGAACTAGC | 3.6 ± 2.8 | 0.2 ± 0.2 |
| mmu-mir-6236-p3_1ss3CG | ATGAACTAGCCCTGAAAAT | 1.5 ± 1.5 | 1.6 ± 1.6 |
| mmu-mir-6236-p3_1ss5CG | GAATGAACTAGCCCTGAAA | 722.5 ± 351.4 | 1354.8 ± 1104.6 |
| mmu-mir-6236-p5_2 | ACTCACCTGCCGAATCAAC | 1.8 ± 1.8 | 0.1 ± 0.1 |
| mmu-mir-6236-p5_3 | CAACTAGCCCTGAAAATGG | 1.8 ± 1.8 | 0.1 ± 0.1 |
| mmu-mir-6236-p5_1ss17GA | AAAATGGATGGCGCTGAAG | 0.2 ± 0.2 | 0.1 ± 0.1 |
| mmu-mir-6236-p5_1ss19GC | CTGAAAATGGATGGCGCTC | 1.4 ± 1.4 | --- |
| mmu-mir-6236-p5_1 | AATCAACTAGCCCTGAAAATGG | 1.8 ± 1.8 | 0.1 ± 0.1 |
| mmu-mir-6240-p5_1ss2GA | CAATGTGATTTCTGCCCAG | 2.2 ± 2.1 | --- |
| mmu-mir-6240-p3_1 | GATTTCTGCCCAGTGCTCT | 79.3 ± 41.8 | 92.4 ± 77.4 |
| mmu-miR-6240_L-4R-4_1ss21AG | AGCATCGCGAAGGCCCGC | 38.9 ± 38.2 | 19.3 ± 12.8 |
| mmu-mir-6240-p3_1ss12AG | CGCGAAGGCCCGCGGCGGG | 4.9 ± 9.9 | 33.3 ± 18.0 |
| mmu-mir-6240-p3_1ss17GT | TGCCCAGTGCTCTGAATGTC | 184.8 ± 83.9 | 235.6 ± 164.7 |
| mmu-mir-6240-p3_1ss1GA | AATGTGATTTCTGCCCAGT | 0.5 ± 0.3 | 0.5 ± 0.5 |
| mmu-mir-6240-p5 | TTCTGCCCAGTGCTCTGA | 1.9 ± 1.8 | 0.4 ± 0.3 |
| mmu-mir-6240-p3_2 | TTTCTGCCCAGTGCTCTG | 79.3 ± 41.8 | 92.4 ± 77.4 |
| mmu-mir-6240-p3_1ss2GA | CAATGTGATTTCTGCCCA | 11.1 ± 8.2 | 3.3 ± 1.7 |
| rno-mir-6320-p3_1ss6AG | AGGCTGAGGCAGGAGGATC | 6.7 ± 6.7 | --- |
| rno-mir-6320-p3_1ss5AG | GGCTGAGGCAGGAGGATC | 12.2 ± 12.2 | --- |
| mmu-miR-6412_R-4_1ss15AT | TCGAAACCATCCTCTGCT | 0.1 ± 0.1 | --- |
| mmu-mir-7050-p5_1ss6GA | AAGGGAGTGAGAGACTCC | 2.4 ± 1.5 | 1.6 ± 1.4 |
| eca-mir-8986b-p5 | CCCGAGACTAGAGTCACATCCTGACA | 203.6 ± 115.6 | 64.6 ± 28.4 |
| eca-mir-8986b-p3_1 | CCCGAGACTAGAGTCACAT | --- | 0.1 ± 0.1 |
| eca-mir-8986b-p3_2 | CCCGAGACTAGAGTCACATC | --- | 0.1 ± 0.1 |
| efu-mir-9226-p5_1ss4AT | TCATGTCCCTGTTCGGGC | --- | 0.7 ± 0.6 |
| efu-miR-9226_R-1 | TCAAGTCCCTGTTCGGGCGCC | 9.9 ± 6.0 | 0.7 ± 0.7 |
| efu-miR-9226_R-4_1ss4AG | TCAGGTCCCTGTTCGGGC | 1.0 ± 0.6 | 7.6 ± 6.0 |
| efu-miR-9226_L-2R-2_1ss4AG | AGGTCCCTGTTCGGGCGC | 29.2 ± 14.3 | 69.4 ± 62.4 |
| efu-miR-9226_R-3_1ss4AG | TCAGGTCCCTGTTCGGGCG | 0.3 ± 0.2 | 0.6 ± 0.6 |
| efu-miR-9277_L-6R+1 | AATCCTGCCGACTACGCC | 3.2 ± 1.7 | 3.1 ± 1.3 |
| efu-miR-9277_L-3R+1 | TCGAATCCTGCCGACTACGCC | 25.2 ± 13.9 | 4.7 ± 2.0 |
| efu-miR-9277_L-3 | TCGAATCCTGCCGACTACGC | 8.0 ± 2.8 | 2.6 ± 2.3 |
| efu-mir-9298-p5_1ss15AG | ACGGGCGGACAGAGGCTC | --- | 0.2 ± 0.2 |
| efu-mir-9341-p5_1ss2TC | TCGCTTCTCGGCCTTTTGGC | 64.8 ± 49.8 | 1.4 ± 0.9 |
| efu-miR-9341_L+3R+1 | GCTTCTCGGCCTTTTGGCTAAGATC | 0.1 ± 0.1 | --- |
| efu-mir-9341-p5_1ss3TC_3 | ATCGCTTCTCGGCCTTTTGGCT | 0.2 ± 0.1 | --- |
| efu-mir-9341-p3_1ss15GC | CTTTTGGCTAAGATCAAGTGTAGTA | --- | 0.1 ± 0.1 |
| efu-mir-9341-p5_2 | GCTTCTCGGCCTTTTGGCTAAGA | 0.1 ± 0.1 | 1.0 ± 0.6 |
| efu-miR-9341_L-2R+4 | TCGGCCTTTTGGCTAAGATCAAG | 0.1 ± 0.1 | --- |
| efu-mir-9341-p5_1 | GCTTCTCGGCCTTTTGGC | 0.1 ± 0.1 | 1.0 ± 0.6 |
| efu-mir-9341-p5_1ss3TN | ATNGCTTCTCGGCCTTTTGGC | 1.2 ± 1.2 | --- |
| efu-mir-9341-p5_1ss3TC_5 | ATCGCTTCTCGGCCTTTTGGCTAA | 0.2 ± 0.1 | --- |
| efu-miR-9341_L+5R-1 | TCGCTTCTCGGCCTTTTGGCTAAGA | 7.2 ± 2.5 | 4.6 ± 1.6 |
| efu-miR-9341_L+6R-1_1 | ATCGCTTCTCGGCCTTTTGGCTAAGA | 281.5 ± 81.2 | 159.3 ± 43.1 |
| efu-miR-9341_L+6R-1_2 | ATNGCTTCTCGGCCTTTTGGCTAAGA | 281.5 ± 81.2 | 159.3 ± 43.1 |
| efu-mir-9341-p3_1ss3TC_1 | ATCGCTTCTCGGCCTTTT | 7.0 ± 4.4 | 8.7 ± 4.5 |
| efu-mir-9341-p5_1ss4TC | CATCGCTTCTCGGCCTTTTGGC | 1.2 ± 1.2 | --- |
| efu-mir-9341-p5_1ss5CT | GCTTTTCGGCCTTTTGGC | 0.6 ± 0.6 | --- |
| efu-mir-9341-p5_1ss3TC_2 | ATCGCTTCTCGGCCTTTTGGC | 0.2 ± 0.1 | --- |
| efu-mir-9341-p5_1ss3TC_4 | ATCGCTTCTCGGCCTTTTGGCTA | 0.2 ± 0.1 | --- |
| ssc-mir-10391-p3 | GTAAACCAGAAAAGGAGGA | --- | 0.3 ± 0.3 |
| bta-miR-11987_L-2_1ss8TA | AGGAAACTCTGGTGGAGGT | 0.1 ± 0.1 | 1.7 ± 1.2 |
| bta-miR-11987_L-1R-1_1ss8TA | GAGGAAACTCTGGTGGAGG | 4.9 ± 2.5 | 3.3 ± 1.3 |
| bta-miR-11987_L-1_1ss8TA | GAGGAAACTCTGGTGGAGGT | 2.0 ± 1.8 | 1.3 ± 0.9 |
| bta-miR-11987_L-2R-1_1ss8TA | AGGAAACTCTGGTGGAGG | 3.0 ± 2.0 | 4.1 ± 1.9 |
| bta-mir-12051-p5_1ss16GC | CGGCGGGGAGAAGAACAAC | 7.7 ± 3.8 | 4.2 ± 2.7 |
| bta-miR-16b_L+2R+1 | GGTAGCAGCACGTAAATATTGGCG | 0.3 ± 0.3 | --- |
| oan-miR-27b-3p_R+2 | TTCACAGTGGCTAAGTTCTGCGT | 0.1 ± 0.1 | --- |
| mmu-miR-146a-5p_R+2 | TGAGAACTGAATTCCATGGGTTAA | 22.4 ± 17.1 | 10.5 ± 5.4 |
| hsa-miR-203a-3p_R-2 | GTGAAATGTTTAGGACCACT | 62.7 ± 14.6 | 105.5 ± 18.2 |
| oan-miR-223-3p_1ss22AT | TGTCAGTTTGTCAAATACCCTT | 2.6 ± 1.6 | 12.3 ± 6.3 |
| hsa-miR-320b_R-1_1ss20CA | AAAAGCTGGGTTGAGAGGGAA | 2.3 ± 1.1 | 1.9 ± 1.2 |
| cgr-miR-326_1ss20GC | CCTCTGGGCCCTTCCTCCACT | 0.2 ± 0.2 | --- |
| bta-miR-342_L-2 | TCACACAGAAATCGCACCCATCT | 1.9 ± 1.9 | --- |
| mmu-miR-344d-3p_L-2R-2_1ss8CG | TATAAGCACTGCCAGACT | --- | 0.7 ± 0.7 |
| bta-miR-378_R+1_1ss6AG | ACTGGGCTTGGAGTCAGAAGGCT | 0.4 ± 0.4 | --- |
| ssc-miR-451_L+4R-1 | GATCAAACCGTTACCATTACTGAGT | 83.3 ± 27.1 | 6.4 ± 4.9 |
| cgr-miR-486-5p_R+3 | TCCTGTACTGAGCTGCCCCGAGGTT | 7.1 ± 2.3 | 4.8 ± 1.7 |
| ppy-miR-1246_L-1 | ATGGGTTTTTGGAGCAGG | --- | 7.0 ± 4.7 |
| hsa-miR-1260b_R+1_1ss9AG | ATCCCACCGCTGCCACCATT | 1.3 ± 0.8 | 0.5 ± 0.5 |
| oan-miR-1386 | CTCCTGGCTGGCTCGCCA | 69.5 ± 62.7 | 8.9 ± 2.6 |
| bta-miR-2284y_R+1_1ss2AG | AGAAGTTCGTTCGGGTTTTTCT | 0.2 ± 0.2 | 0.5 ± 0.4 |
| bta-miR-2285f | AAAACCTGAATGAACTTTTTGG | 1.1 ± 1.1 | 0.8 ± 0.6 |
| bta-miR-2285ba_1ss7TC | AAACCCCGAACGAACTTTTTGG | 1.1 ± 1.0 | 0.9 ± 0.5 |
| bta-miR-2478_L-2 | ATCCCACTTCTGACACCA | 8.6 ± 5.5 | 3.5 ± 3.1 |
| bta-miR-2904_L+6R+1 | CGCCGCGGGAGCCTCGGTTGGCCTCG | 279.5 ± 90.6 | 774.1 ± 322.1 |
| hsa-miR-3168_R+1_1ss14AC | GAGTTCTACAGTCCGACA | --- | 0.7 ± 0.5 |
| hsa-miR-4454 | GGATCCGAGTCACGGCACCA | 3.3 ± 3.0 | 0.1 ± 0.1 |
| hsa-miR-5100_L-3R-1 | AGATCCCAGCGGTGCCTC | 0.9 ± 0.6 | --- |
| mmu-miR-5106_R-4_1ss1AG | GGGTCTGTAGCTCAGTTGG | 45.9 ± 25.5 | 14.9 ± 8.1 |
| mmu-miR-5119_R-1_1ss5TG | CATCGCATCCTGGGGCTG | 8.9 ± 4.4 | 1.5 ± 0.7 |
| mmu-miR-6239_R-2_1ss6TG | TAGCGGTGGATCACTCGG | --- | 2.4 ± 1.7 |
| ssc-miR-7134-5p | ATGTCCGCGGGTTCCCTATCC | 0.6 ± 0.6 | 1.2 ± 0.9 |
| hsa-miR-7977_1ss6AG | TTCCCGGCCAACGCACCA | 21.8 ± 16.1 | 2.3 ± 0.9 |
| efu-miR-9226_L-2R-1_1ss4AG | AGGTCCCTGTTCGGGCGCC | 242.2 ± 177.6 | 174.9 ± 119.8 |
| efu-miR-9277_L-3_1ss7AT | TCGTATCCTGCCGACTACGC | 0.2 ± 0.2 | 2.8 ± 2.8 |
| bta-miR-11980_R-1_1ss4CG | AGGGAACGGGCTTGGCGGA | 6.7 ± 6.1 | 15.4 ± 10.0 |
| mmu-let-7j_1ss8TG | TGAGGTAGTAGTTTGTGCTGTTAT | 2.0 ± 0.9 | 3.5 ± 1.0 |
| PC-5p-25584_101 | ATGATCTGGCGGCGCTGGCC | 15.1 ± 4.0 | --- |
| PC-3p-95779_15 | CGTGGCCGGCGCTGTCAGTTC | 8.6 ± 6.0 | --- |
| PC-5p-57646_33 | GTCACCAGAAACTGTAGAGG | 1.6 ± 0.9 | --- |
| PC-5p-61642_30 | ACTGCACAGTGGACGCGAG | 0.8 ± 0.8 | 0.8 ± 0.8 |
| PC-5p-23321_113 | GAACAATGTAGATAAGGGA | 5.3 ± 4.2 | 13.3 ± 11.6 |
| PC-3p-79650_20 | GTGCTTCCCTGACATGTT | 0.3 ± 0.3 | 0.1 ± 0.1 |
| PC-3p-47343_45 | TTTCCCCTCAGCTCCACCA | 7.3 ± 4.8 | --- |
| PC-5p-77439_21 | GAGAACAAGAATTCCCGACT | 2.3 ± 2.0 | 0.1 ± 0.1 |
| PC-5p-213458_5 | CGAAGTGGAGAAGGGTTCC | 3.0 ± 3.0 | --- |
| PC-5p-141956_7 | TCTGTGAACCGGCCTGTGT | 2.7 ± 2.7 | --- |
| PC-3p-59071_32 | GGCAGAGATGCGTAGCTGA | 0.3 ± 0.3 | 1.6 ± 1.3 |
| PC-3p-27917_91 | TTCCACCGCTTTCCCGTGG | 11.5 ± 8.0 | 1.8 ± 1.8 |
| PC-3p-46360_46 | CAGGTCCTGGTACGCCCAC | 16.6 ± 13.3 | 6.2 ± 6.2 |
| PC-5p-146681_7 | AGAGAAGAGAACCCAGGGAAT | 0.7 ± 0.7 | --- |
| PC-3p-159155_6 | GCAGGAAAAGAAATCAACC | 3.6 ± 3.6 | --- |
| PC-5p-136439_7 | GGGGGCGTGGAGGCGGGGCGG | 4.3 ± 4.3 | --- |
| PC-3p-84665_18 | GGCGGGAGCCCCGGGGAGA | --- | 3.1 ± 3.1 |
| PC-5p-18152_150 | AGGCGCTTGAGAGAACTC | 32.8 ± 25.2 | 9.0 ± 3.3 |
| PC-5p-87783_17 | TTGCTAGTTGTCGGGCTGC | 2.1 ± 1.8 | 0.6 ± 0.6 |
| PC-5p-42711_52 | CCCGCGAGGGGGCGGGGCGGGG | 1.2 ± 0.8 | 4.6 ± 2.6 |
| PC-3p-165292_6 | CTCAGTGGCAAAGGAAGTG | 0.9 ± 0.9 | --- |
| PC-3p-76207_22 | TGAAGGGTTTGATCCTGGCT | 0.5 ± 0.5 | 0.3 ± 0.3 |
| PC-3p-169805_6 | TTCCTGGGCATTGTTGAGCATGGC | --- | 1.9 ± 1.9 |
| PC-5p-133897_8 | CCTGCATGTTAGTCTTCTGTTCTGA | 5.7 ± 5.7 | 3.4 ± 3.1 |
| PC-5p-203717_5 | TGGGGCTGTAGCTGGTCC | 1.5 ± 1.5 | --- |
| PC-5p-31311_79 | AGTCCTGTCGCCCCTGCCA | 8.0 ± 5.4 | 0.9 ± 0.9 |
| PC-5p-174947_5 | GGAAGAGATAAACGCTGAA | 2.0 ± 2.0 | --- |
| PC-5p-84246_18 | GCCCCTGCATTCGAAAGTGACC | --- | 3.3 ± 3.3 |
| PC-3p-10468_272 | TCGCGGCAGCGGGATGAGG | 12.0 ± 6.1 | 8.1 ± 2.9 |
| PC-5p-121523_10 | CTCGGGGAGAAGAAACAA | 1.0 ± 1.0 | --- |
| PC-3p-103619_13 | GGCTGGGTCGGTCGGGCT | 4.2 ± 3.6 | 2.5 ± 1.7 |
| PC-3p-135113_8 | CCCGGTCGCTGCATTGGA | 15.2 ± 15.2 | --- |
| PC-5p-98622_14 | AAGGGATTGGCGGAGTCTC | 0.8 ± 0.6 | 0.2 ± 0.2 |
| PC-5p-19513_139 | AGGACAGCCCAGAATCAAGAGATG | 57.1 ± 54.5 | 4.9 ± 4.0 |
| PC-3p-70994_24 | AATTAGTGCATACGAAACCACT | 1.6 ± 0.9 | --- |
| PC-5p-71096_24 | CCGGGTCAGGTTTGCAGGCGC | 2.2 ± 1.8 | --- |
| PC-3p-144487_7 | TTCCCGTGGAACTCGGGAG | --- | 1.2 ± 1.2 |
| PC-5p-48412_43 | CTTGACCTCGGATCAGGTA | 4.7 ± 3.7 | 7.5 ± 6.5 |
| PC-5p-25925_99 | AGAGAGAGTTCAAGAGGGC | 9.3 ± 9.1 | 9.9 ± 4.0 |
| PC-5p-29246_86 | AATTCAACCAAGCGCGGG | 0.2 ± 0.2 | 38.3 ± 38.3 |
| PC-3p-62295_30 | GACGCCTGCCCGGTGCTGG | 2.0 ± 1.3 | 1.4 ± 1.1 |
| PC-3p-191609_5 | GCTGGGGATAGAGCATTGCA | --- | 1.4 ± 1.4 |
| PC-5p-27749_92 | GAGTCGCTGAGCGTCAGCC | --- | 89.0 ± 89.0 |
| PC-3p-33463_72 | ATGTTTGTTTCAGTGTGTTT | 0.1 ± 0.1 | --- |
| PC-5p-77448_21 | AGATTGAGAGAGCCTGCTC | 4.8 ± 4.5 | 0.5 ± 0.3 |
| PC-3p-104919_12 | GCCCTGAGGCTTGGAAGGCTC | --- | 2.1 ± 2.1 |
| PC-3p-53623_37 | TTAATAAAAACACAGCACTC | 3.2 ± 2.8 | 0.2 ± 0.2 |
| PC-3p-16382_168 | GAAGCAGGGACGCCAGTTC | 10.2 ± 5.2 | 7.3 ± 2.7 |
| PC-5p-99552_14 | CTGAAGGACGGTTACTAGC | 3.1 ± 3.1 | --- |
| PC-3p-82240_19 | GAGTGTAGGTGTTGAGGAT | 2.3 ± 2.3 | --- |
| PC-3p-11725_240 | CCTGAGTGTCGTACTCCC | 23.5 ± 19.3 | 4.7 ± 1.4 |
| PC-3p-4468_648 | AATCCCTTCGCCCGCTCCA | 40.3 ± 12.2 | 23.5 ± 4.0 |
| PC-3p-75969_22 | CGGCGGCGGTTCTCCGGC | 3.0 ± 3.0 | 5.8 ± 3.9 |
| PC-3p-85929_18 | TGACCAAGGGTTCCTGGGC | 0.4 ± 0.4 | --- |
| PC-3p-10717_265 | GAGGCTATGAAACCGGAAC | 26.5 ± 24.8 | 72.3 ± 64.5 |
| PC-3p-91172_16 | AAGGGGTGGGCGAGGTCC | --- | 2.7 ± 2.7 |
| PC-3p-3926_729 | GAGAACTCGGGAGAAGGAAC | 430.0 ± 380.1 | 23.3 ± 14.5 |
| PC-3p-42375_52 | CCAGACTCGGCCTCGGGGT | --- | 15.0 ± 15.0 |
| PC-5p-123328_10 | TGAGATAAGCGCTGAAAGC | 2.8 ± 2.4 | 0.2 ± 0.2 |
| PC-5p-39996_57 | AAGAGATAAACGCTGAAAG | 3.9 ± 2.9 | 1.8 ± 0.9 |
| PC-5p-39532_58 | ATCAGGGCTGGCTCCGGGA | 12.0 ± 9.2 | --- |
| PC-3p-219880_5 | TGCAGGGTCCGGTGCGCCC | --- | 1.4 ± 1.4 |
| PC-3p-38202_60 | GTGAATGTGTTCCTGAGCC | 6.0 ± 4.5 | 0.3 ± 0.3 |
| PC-5p-89311_17 | TTCCCCGGGGCCGGGCCAG | --- | 10.1 ± 10.1 |
| PC-3p-191352_5 | AATGGGAGGTAAATTTCTT | 3.0 ± 3.0 | --- |
| PC-3p-56235_35 | CTGCAGATTTGAGGAAGCC | 4.3 ± 4.1 | 0.9 ± 0.7 |
| PC-3p-190284_5 | CGCTCTCGGGCGCCGCCTG | 3.0 ± 3.0 | --- |
| PC-3p-84476_18 | GATAAAAGTAGTGAAAAAC | 3.2 ± 2.8 | --- |
| PC-3p-82295_19 | TGGGAAGGCATAGACAGCT | 2.0 ± 2.0 | 1.0 ± 0.7 |
| PC-3p-31096_80 | TTAGGTAGAGTGTGATAGCGTCG | 0.6 ± 0.6 | 3.8 ± 2.2 |
| PC-5p-88802_17 | AGTTATGATTTTGGCATGGT | 0.8 ± 0.6 | --- |
| PC-5p-7443_389 | ATGGAACTTCAGACAACAGGGT | 29.6 ± 7.7 | --- |
| PC-3p-157991_6 | TCTCCTGTCTATTCCTTCGTGGGC | --- | 1.0 ± 1.0 |
| PC-3p-44701_49 | GATTCTGCCCCTGGCCACCA | 0.5 ± 0.5 | 1.8 ± 1.0 |
| PC-3p-41523_54 | CCCTGAACTAGGAGTCTGGAGT | 1.3 ± 0.6 | 2.6 ± 1.4 |
| PC-3p-129291_9 | CCTATGAGCCGTGCTTTT | 1.6 ± 1.6 | --- |
| PC-3p-90989_16 | CCCCCATCACCGAGCTTGAC | --- | 2.7 ± 2.0 |
| PC-5p-86185_18 | TAATAGATCGTGAGGCTT | 0.9 ± 0.6 | 0.1 ± 0.1 |
| PC-3p-118085_11 | TCGAATCCCACCCCATCTGCC | 0.4 ± 0.4 | --- |
| PC-3p-131815_8 | AGGTACAGAGAGACGGTCC | 0.8 ± 0.8 | --- |
| PC-5p-217520_5 | CTGCGTCCTCTGCGGCTCT | 2.0 ± 2.0 | --- |
| PC-5p-21752_123 | GGGGGTAGCGACTGTTTA | 4.8 ± 2.8 | 2.8 ± 1.3 |
| PC-3p-26295_98 | GTGGTATCCTGAGTAGGGC | 5.3 ± 4.5 | 2.3 ± 1.2 |
| PC-3p-67558_26 | GGGAGTCGGGTTCAGATCCC | 0.6 ± 0.6 | 2.3 ± 1.4 |
| PC-3p-70811_24 | GAGAGGTATGTGGAACTCC | 1.2 ± 0.9 | --- |
| PC-3p-75721_22 | CGACTCCTGCCTGGCTCGGT | 0.6 ± 0.4 | --- |
| PC-3p-66418_27 | GAAGACTGAAGTGGAGAA | 1.1 ± 0.5 | 0.5 ± 0.3 |
| PC-5p-118259_11 | AAAGGGACAGTCGATGGAC | 2.0 ± 2.0 | --- |
| PC-5p-135504_8 | GGAACAATTTTTGGCTGTCT | --- | 4.0 ± 4.0 |
| PC-5p-150413_6 | TTCAGGAGGCTTGCTGGACT | 2.5 ± 2.5 | --- |
| PC-5p-22856_116 | AAGTCCTCCCAGGCCCACCA | 3.9 ± 2.4 | 1.8 ± 1.0 |
| PC-5p-76549_21 | ATGTCCCTGTTGCTGTATC | 5.2 ± 4.3 | 0.3 ± 0.3 |
| PC-3p-68332_26 | CAGCCTCGATTCTGTTTC | 0.3 ± 0.3 | 0.4 ± 0.4 |
| PC-5p-28051_90 | GTGGAGTGATTTGTCTGGTT | 46.2 ± 31.0 | 74.6 ± 68.3 |
| PC-3p-195244_5 | ATATAGGGCGTGTGGAGGGAA | 3.0 ± 3.0 | --- |
| PC-3p-103987_13 | GACACCAAGGGGCCTTCT | --- | 0.1 ± 0.1 |
| PC-3p-107601_12 | CCGCCGCCTGAGGTCCAC | 5.8 ± 4.4 | --- |
| PC-3p-48170_44 | GACGGATCTCTGGTGGACC | 0.4 ± 0.3 | 0.8 ± 0.8 |
| PC-3p-32331_76 | CTTCGGGCCATGCACCTC | --- | 24.5 ± 24.5 |
| PC-5p-116728_11 | GGGTCTGACGCCTGCCCGG | --- | 0.1 ± 0.1 |
| PC-3p-106495_12 | TAGGCCAGAACTGGAAGT | 4.3 ± 4.1 | --- |
| PC-5p-67320_26 | TTGGCTCTGAGGGCTGGGCACG | 0.3 ± 0.3 | --- |
| PC-5p-3076_932 | AAGGGCCATCGCTCAACGG | 205.8 ± 83.8 | 83.7 ± 16.0 |
| PC-3p-221689_5 | GAGAGAGGTATGTGGAACT | 0.8 ± 0.8 | 0.5 ± 0.5 |
| PC-3p-92213_16 | CAGAAGTGCGAATGCTGGC | 2.5 ± 2.5 | --- |
| PC-5p-71420_24 | ATCCTGAGTAGGGCGGGGC | 0.3 ± 0.3 | 0.2 ± 0.2 |
| PC-5p-110848_11 | TGGTGAAAGTCCAGTAGTT | --- | 2.3 ± 1.7 |
| PC-5p-99536_14 | AAATCTGGTTCCTGGCACCA | 0.6 ± 0.6 | --- |
| PC-3p-93652_15 | TGAAGAGACATGAGAGGTGTA | 0.4 ± 0.4 | 2.0 ± 1.3 |
| PC-5p-9157_315 | AAGTCGTAACAAGGTTTCC | 135.7 ± 80.8 | 30.6 ± 6.3 |
| PC-5p-7060_410 | GATTCCCTCTGCCCCTGCCA | 34.4 ± 20.9 | 3.3 ± 3.0 |
| PC-3p-53744_37 | AGGGCTGGTGGCTTTCTGTGG | --- | 7.2 ± 7.2 |
| PC-3p-14431_193 | CCCCGAGGGGCTCTCGCT | 25.3 ± 18.4 | 56.9 ± 44.7 |
| PC-3p-95567_15 | GCCTCCGTCCCCTCCGCCC | 3.6 ± 3.6 | 1.6 ± 1.6 |
| PC-5p-65406_27 | ACAATCAGGAGGTTTGCTT | 3.8 ± 3.2 | 0.4 ± 0.3 |
| PC-5p-122000_10 | AAATAGCTCAGTGCCAGGTT | --- | 0.2 ± 0.2 |
| PC-5p-31728_77 | TGCGGCTGGAACACCTCCT | 6.5 ± 4.6 | 1.3 ± 0.7 |
| PC-3p-126208_10 | AGGGTGAGGCCCAGGAATCTGCATTC | --- | 1.7 ± 1.7 |
| PC-5p-80292_20 | AATAAAAACACAGCACTCTG | 3.5 ± 2.8 | 0.2 ± 0.2 |
| PC-5p-203794_5 | AGCTTGAGAGGATCTGTCC | 0.8 ± 0.8 | --- |
| PC-3p-102485_13 | AAATTTGAAATCTGGTTCC | 2.0 ± 2.0 | --- |
| PC-3p-82438_19 | TCGTAAGATTAAAACTCAA | 2.5 ± 2.5 | --- |
| PC-5p-12214_230 | GAGGTTCCAGGAAATAGCT | 1.7 ± 0.8 | 2.8 ± 2.0 |
| PC-3p-15114_184 | AGGGGAGTGAAATAGATC | 15.0 ± 12.5 | 6.7 ± 2.6 |
| PC-3p-170275_6 | CCCCTCAGCAGCAGCACTGGCACCCC | --- | 1.0 ± 1.0 |
| PC-5p-20665_130 | CACCTGGTATCCCTCCCC | 12.3 ± 7.6 | 2.9 ± 1.6 |
| PC-5p-52973_38 | AGTGACTAAGAGCATGTGG | 10.4 ± 9.7 | 0.2 ± 0.2 |
| PC-3p-35248_67 | GCGACCACGTTCCCGTGG | 14.6 ± 11.4 | 2.5 ± 2.5 |
| PC-5p-154777_6 | GAGCTGTGGGTAGGGGTGA | 0.9 ± 0.9 | --- |
| PC-5p-55110_36 | GTGGGGAGTTTGGCTGGG | 3.6 ± 2.4 | 0.6 ± 0.6 |
| PC-3p-51225_40 | TGCGATGTGGGGACGGAGA | 0.9 ± 0.6 | 0.4 ± 0.4 |
| PC-5p-15621_177 | GAGGACCGGGATGGACGT | 8.5 ± 3.8 | 8.8 ± 4.0 |
| PC-5p-43707_50 | AAATGATGTGTAGGATAGGT | 5.3 ± 4.5 | 1.2 ± 0.8 |
| PC-5p-50895_40 | AGGTGGACTGGTAGAGTA | 2.9 ± 2.4 | 1.4 ± 1.1 |
| PC-5p-4250_682 | ACAGGATCTGTCCCTAGT | 26.4 ± 10.2 | 20.5 ± 6.0 |
| PC-5p-7229_401 | CCGTGGACGGTGTGAGGCC | 22.4 ± 14.2 | 29.2 ± 10.7 |
| PC-5p-108274_12 | CTGGGGAATCCCATGGCCT | --- | 3.5 ± 3.5 |
| PC-5p-3896_735 | CCTGAGCTAATGACAAAGG | 111.8 ± 73.9 | --- |
| PC-3p-209347_5 | GGCATCTTAGGGGACTGGGAC | 3.0 ± 3.0 | --- |
| PC-5p-106110_12 | CTGAAGAGTCCACTTTCC | 1.0 ± 0.7 | --- |
| PC-3p-44065_50 | GGTGGAGGAAAGGCTCTG | 7.2 ± 5.4 | --- |
| PC-5p-32115_76 | GCGGAGACGTCGGCGGGGG | --- | 24.2 ± 24.2 |
| PC-5p-87767_17 | CCTTCAGGCGCCTGCAGCCCTCT | 1.0 ± 0.6 | --- |
| PC-3p-41449_54 | CTGTAGCTTTGTATTGGAC | 13.8 ± 12.1 | 2.2 ± 1.1 |
| PC-5p-211407_5 | ACAGGCTGCCCCATCCGAT | 0.8 ± 0.8 | --- |
| PC-5p-86846_17 | TTGAGTGCGGCAGAGGGGGA | 2.0 ± 2.0 | 0.2 ± 0.2 |
| PC-5p-6087_476 | AGAGAACTCGGGAGAAGGAA | 146.9 ± 121.2 | 36.7 ± 32.9 |
| PC-3p-123545_10 | CACGATGAGACAGAGGACG | 2.2 ± 2.0 | --- |
| PC-5p-152421_6 | GGTCCCCTTAGCTCCACCA | 0.9 ± 0.9 | --- |
| PC-5p-20853_129 | TCTGGAGAAAGGGAGAGGAAG | 39.0 ± 17.3 | --- |
| PC-3p-87203_17 | AAGTGATCTATCCATGGCC | 0.3 ± 0.3 | 0.2 ± 0.2 |
| PC-3p-203435_5 | CAGAGACCAGGGGGAAGCG | 0.8 ± 0.8 | --- |
| PC-3p-7345_394 | GAGGTGAAATTCGTAGATA | 34.0 ± 18.8 | 21.9 ± 6.0 |
| PC-5p-40997_55 | AATCCCCCTCTGTCCGCCA | 11.2 ± 7.9 | 2.5 ± 2.5 |
| PC-3p-111009_11 | AACAGATAAAAGGTACTCC | 2.0 ± 2.0 | 0.1 ± 0.1 |
| PC-5p-54437_36 | GAACTTAAGAGTTTGATCCT | 0.5 ± 0.5 | 0.1 ± 0.1 |
| PC-5p-53467_37 | CGCCGGGCGGCGGGCCGCCA | 2.3 ± 2.3 | --- |
| PC-5p-77510_21 | CGGTGTCGCTCAACGGATA | 3.2 ± 3.2 | --- |
| PC-5p-28019_91 | AACAGATCTGTGTAGGATA | 5.9 ± 5.3 | 1.1 ± 0.6 |
| PC-3p-123000_10 | GGCGCTGCAAGATTGAGAG | 0.2 ± 0.2 | 0.2 ± 0.2 |
